# Discovering High Entropy Alloy Electrocatalysts in Vast Composition Spaces with Multiobjective Optimization

**DOI:** 10.1021/jacs.3c14486

**Published:** 2024-03-11

**Authors:** Wenbin Xu, Elias Diesen, Tianwei He, Karsten Reuter, Johannes T. Margraf

**Affiliations:** † Fritz-Haber-Institut der Max-Planck-Gesellschaft, Berlin D-14195, Germany; ‡ Lawrence Berkeley National Laboratory, Berkeley, California 94720, United States; § Yunnan Key Laboratory for Micro/Nano Materials & Technology, National Center for International Research on Photoelectric and Energy Materials, School of Materials and Energy, Yunnan University, Kunming 650091, China; ∥ Bavarian Center for Battery Technology (BayBatt), University of Bayreuth, Bayreuth D-95447, Germany

## Abstract

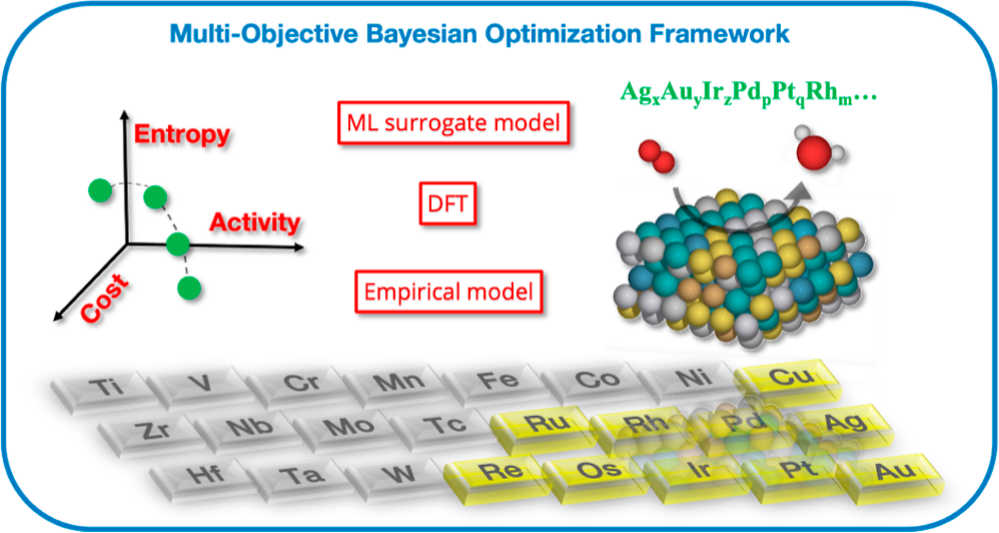

High entropy alloys (HEAs) are a highly promising class of materials for electrocatalysis as their unique active site distributions break the scaling relations that limit the activity of conventional transition metal catalysts. Existing Bayesian optimization (BO)-based virtual screening approaches focus on catalytic activity as the sole objective and correspondingly tend to identify promising materials that are unlikely to be entropically stabilized. Here, we overcome this limitation with a multiobjective BO framework for HEAs that simultaneously targets activity, cost-effectiveness, and entropic stabilization. With diversity-guided batch selection further boosting its data efficiency, the framework readily identifies numerous promising candidates for the oxygen reduction reaction that strike the balance between all three objectives in hitherto unchartered HEA design spaces comprising up to 10 elements.

## Introduction

High entropy alloys (HEAs) constitute an emerging class of materials that holds great promise for novel catalysts with enhanced properties, including activity, selectivity, stability, and cost-effectiveness.^1−5^ The strong interest in HEAs for catalysis stems from their almost continuous distribution of adsorption energies due to the entropically stabilized, highly disordered local arrangement of their constituting elements, giving rise to a wide range of surface compositional motifs. In contrast to the discrete binding energies of conventional (unary, intermetallic, core/shell, etc.) alloys, these continuous distributions are believed to be better tunable to match the “optimal” binding strengths in the sense of the classic Sabatier principle.^6^ “Tunable” here refers to the number and type of constituting elements and their respective molar fractions, which in total spans a high-dimensional design space. In this respect, intense efforts have recently been undertaken to efficiently explore the vast HEA composition spaces by means of high-throughput experiments^7−9^ and theory-guided screening studies.^10−16^

Despite recent advances, these efforts are restricted to relatively low-dimensional compositions, barely scratching the surface of the vast design space available for this class of materials. For instance, recent theoretical^13,15,17−22^ and experimental^7,8,23^ studies limit the optimization to 5-element HEA spaces, and the selected constituent five elements are primarily based on experimental observations or chemical intuition. This hinders the understanding of higher-dimensional landscapes and limits exploration to regions that have been extensively experimentally tested. Nonetheless, thanks to ongoing endeavors toward alloying a higher number of elements, it has become evident that higher-dimensional HEAs composed of 6,^24−27^ 8,^28−30^ 10,^31^ 12,^32^ 14,^33,34^ and even 21^35^ elements can be successfully synthesized. To date, these higher-dimensional spaces remain largely unexplored, and their growing complexity and associated combinatorial explosion call for the development of advanced search methods and optimization frameworks.

In terms of optimization methodologies, single-objective Bayesian optimization (BO) combined with experimental tests or machine learning (ML) surrogate models for evaluation of the catalyst activity has been employed with promising results. This method has proven effective in significantly reducing the number of evaluations required, typically down to around 50, to identify the globally optimal composition for a 5-element HEA space.^21,23^ That said, it is important to note that this method prioritizes the catalytic activity as the sole target property for optimization. Consequently, the top-ranking candidates identified in the searched HEA space often turn out to be previously known binary alloys.^11,21^ For such binary alloys, the formation of a single solid solution is less likely due to missing entropic stabilization. Instead, they are more likely to form ordered phases with a few discrete binding energies. This contradicts the assumption that HEA catalysts benefit from featuring a complex distribution of binding energies.

In our view, the discovery of effective HEA catalysts therefore necessitates considering multiple properties simultaneously. For instance, a good catalyst must not only exhibit high catalytic activity but also demonstrate qualities such as sufficient cost-effectiveness, stability, and synthesizability. A noteworthy example explored the Pareto front of catalytic activity and element scarcity on a uniform 5 at% grid of the AgIrPdPtRu HEA composition space. This demonstrated that by considering the scarcity of the constituent elements, the focus of catalyst discovery shifts from binary compositions with high catalytic activity to more complex ones, including abundant elements.^36^ Additionally, multifunctional catalysts need to promote multiple catalytic reactions (i.e., as target properties),^27,37^ which is crucial for cascade reactions such as electrocatalytic CO_2_ reduction.

Besides its focus on a sole target property, the single-objective BO in these studies also places excessive emphasis on a single global optimum within the explored 5-element space,^11,21,38,39^ which is not directly applicable to higher-dimensional scenarios. A recent experiment conducted in a 6-element HEA space has demonstrated the existence of multiple optimal solutions.^26^ Moving toward even higher dimensions is expected to exacerbate this problem. Furthermore, in the context of multiobjective optimization,^12,40,41^ the notion of a single optimum is conceptually flawed. Instead, candidates situated on the Pareto front—i.e., not dominated by any other alternative—are equally important, and the ultimate choice depends on the specific user-defined trade-off among the target properties.^42^

In order to dive into higher-dimensional composition space with an unbiased choice of constituent elements and discover novel catalysts with multiple desired properties, we here present a data-efficient multiobjective BO framework that allows navigating different trade-offs along the Pareto front. As a showcase, we focus on the discovery of HEA electrocatalysts for the oxygen reduction reaction (ORR) with compositions comprised of up to 10 elements. This translates to about 4.3 × 10^12^ possible HEAs and is thus 6 orders of magnitude larger than a 5-element space (4.6 × 10^6^), when assuming a molar fraction step size of 1%. We perform simultaneous optimizations of catalytic activity, cost-effectiveness, and mixing entropy in this vast design space and suggest a number of promising HEAs. Our findings highlight the promise of ML surrogate model-driven multiobjective optimization for material discovery.

## Results and Discussion

### Multiobjective Bayesian Optimization Framework

**Figure 1:**
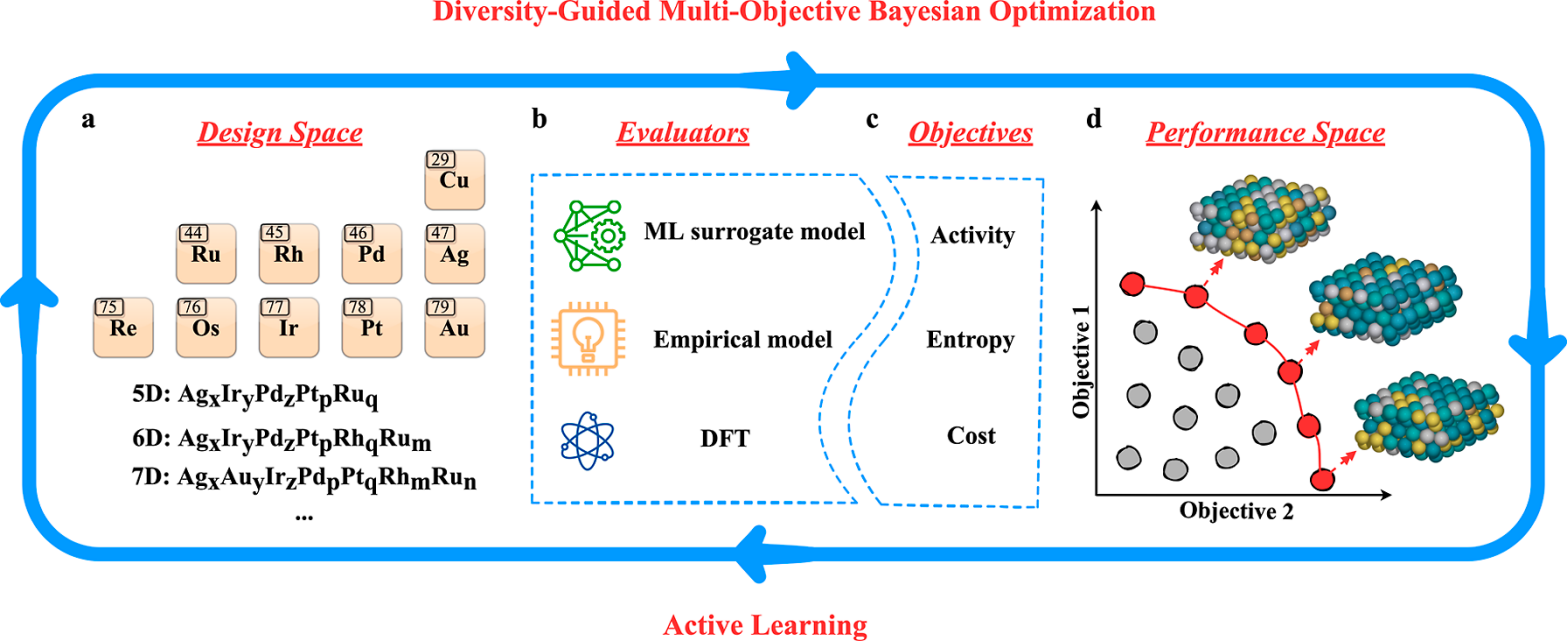
Schematic illustration of the framework for discovering HEA electrocatalysts for ORR. (a) 10-element design space with a molar fraction step size of 1%. The possible HEAs can be made up of arbitrary compositions. (b) Evaluators used to compute the values of the targeted objectives. The ML surrogate model refers to the graph neural network (GNN) regression model for adsorption enthalpy prediction. The term “empirical model” refers to the current density modeling and the computation of correlated mixing entropies. (c) Three target objectives, catalytic activity, mixing entropy, and cost-effectiveness, are considered in this work. (d) Learned continuous Pareto front in performance space. The term “active learning” refers to the update of the surrogate model during Bayesian optimization and the iterative expansion of the Pareto front in the performance space.

The multiobjective BO framework depicted in Figure 1 is built upon a customized diversity-guided approach (see Section S4 of the Supporting Information) that operates on batches of evaluated samples in each iteration to improve the Pareto front.^43^ The built-in batch selection strategy takes into account the diversity of both the design and performance space, allowing for efficient and effective sampling. By using this framework, our goal is to discover HEA electrocatalysts for the ORR with multiple targeted objectives from a vast design space with up to 10 elements (Ag, Au, Cu, Ir, Os, Pd, Pt, Re, Rh, and Ru), including 3d, 4d, and 5d transition metals that are potentially significant for the ORR. We consider this 10-element space and all of its subspaces by taking a molar fraction step size of 1% for each constituent element. As mentioned above, this leads to about 4.3 × 10^12^ possible HEA materials and correspondingly a tremendously challenging discovery task. The performance space we explore spans up to three objective dimensions, i.e., catalytic activity, cost-effectiveness, and mixing entropy, and we aim at identifying a continuous Pareto front that offers the ability to select materials with different trade-offs of interest.

The design space is mapped to the performance space through evaluators involving an ML surrogate model based on first-principles density functional theory (DFT) data, as well as empirical models. Specifically, the ML surrogate model in this work refers to a GNN regression model for adsorption enthalpy prediction. However, using high-throughput experimental tests as evaluators would also be possible, aligning well with the built-in batch selection scheme of our multiobjective BO framework: Independent experimental trials can be carried out in parallel without delay. Ultimately, the entire framework proceeds in an active-learning fashion, for which the Pareto front is progressively expanded by incorporation of suggested batch samples. A detailed description of the diversity-guided multiobjective BO method, the GNN model, DFT calculations, and the empirical model can be found in the Supporting Information.

### ML-Enabled Catalytic Activity Prediction

The aim is to develop a model capable of predicting the catalytic activity for HEAs with arbitrary compositions. However, conventional methods encounter difficulties in assessing the catalytic activity for two main reasons. First, the statistical nature of HEAs results in a vast number of possible active sites per surface. Calculating the adsorption enthalpies, which serve as key activity descriptors, for all of these sites using standard DFT calculations is computationally prohibitive. This motivates the use of a computationally inexpensive ML regression model; given the vast 10-element design space targeted in this work, the ML regression model must exhibit reliable extrapolation capabilities to handle unknown materials. Second, even if the adsorption enthalpies were known, the methods typically used for surface kinetics modeling, such as mean-field approximation^44,45^ or Kinetic Monte Carlo methods,^46,47^ face challenges when confronted with the inherent complexity of HEAs. An alternative approach capable of addressing these challenges is needed.

**Figure 2:**
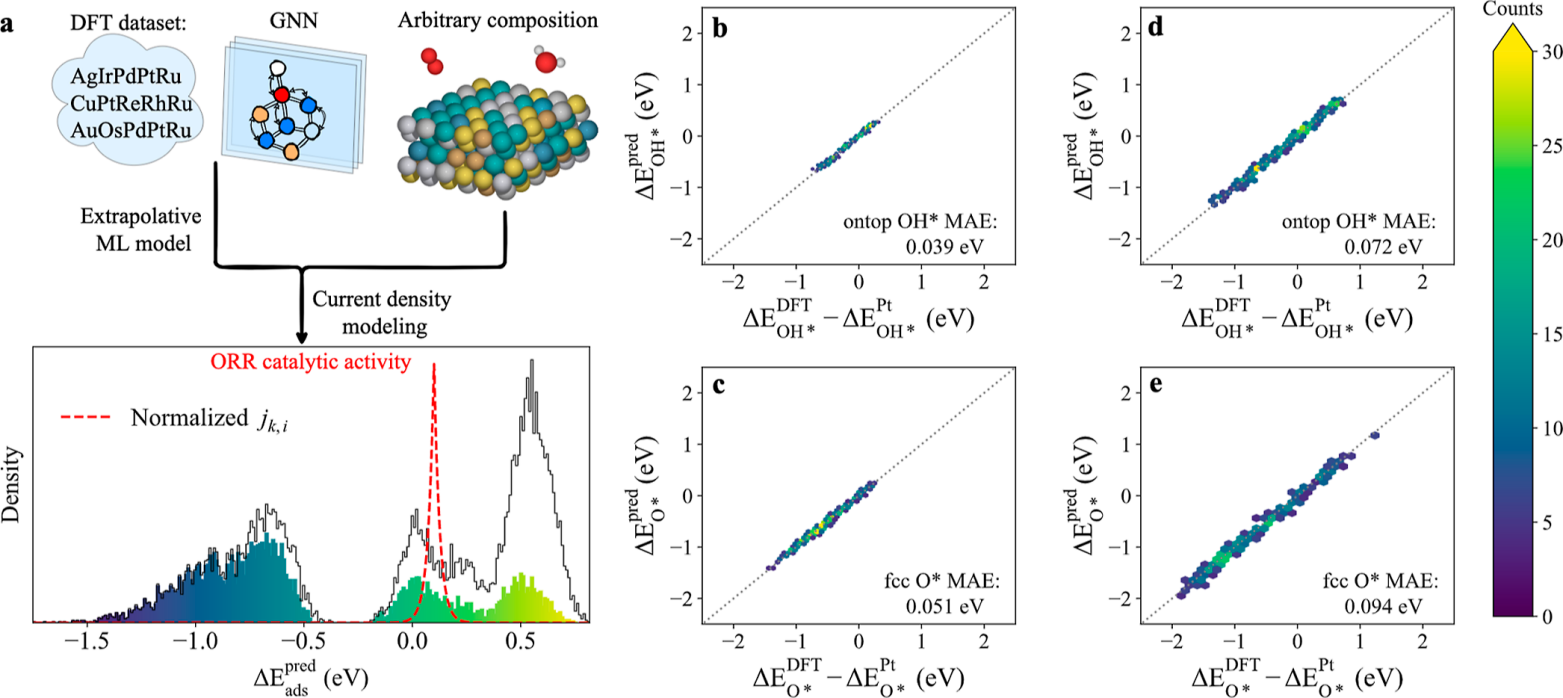
(a) Schematic illustration of ML-enabled catalytic activity prediction. The key components include an extrapolative ML model and current density modeling, wherein the former is a GNN regression model trained on our established DFT data set. The resulting plot related to catalytic activity presents both gross (gray outlines) and net (color-filled bars) adsorption enthalpy distributions, along with the normalized current density (red dotted line). (b–e) Parity hexbin plots of DFT-calculated versus ML-predicted adsorption enthalpies for out-of-domain predictions, including (b) on-top OH*, (c) fcc hollow O* for the composition-diversity data set, (d) on-top OH*, and (e) fcc hollow O* for the component-diversity data set. The color bar indicates the number of data points contained in each hexbin.

To this end, as demonstrated in Figure 2a, we employ an extrapolative GNN model based on a variant of gated graph convolutional networks^48^ that has recently been developed for predicting 5-element HEA catalysts^11^ (see also the software repository^49^). The model is trained on a new HEA data set including the 5-element HEA spaces AgIrPdPtRu, AuOsPdPtRu, and CuPtReRhRu (see Section S1 of the Supporting Information) to enable the prediction of adsorption enthalpies on arbitrary HEAs within the 10-element composition space. The consideration of both O* and OH* in the training data set is due to the failure of widely used binding energy linear scaling relations and Brønsted–Evans–Polanyi relations in describing HEAs,^9,50^ so that going beyond a single descriptor, i.e., O* or OH* alone, is required to describe the ORR mechanism. Note that the ML model employed here is a “discrete” approach, focusing solely on learning the local minima of the potential energy surface. This approach takes a graph representation (without 3D geometry information) of the initial state and predicts the relaxed adsorption enthalpy. It is fundamentally distinct from a “continuous” approach, i.e., an ML interatomic potential, which learns the entire landscape of the potential energy surface. In contrast, the latter approach is trained to relax the initial structure and, thereby, predict both the relaxed structure and adsorption enthalpies. Subsequently, we integrate this GNN model with a heuristic current density modeling technique that mimics surface coadsorption of O* and OH* for ORR to obtain their net adsorption enthalpy distributions and associated catalytic activity, i.e., average current density (see Section S5 of the Supporting Information). O* and OH* are considered to be adsorbed on their favorable fcc hollow and on-top sites, respectively.

To train the GNN model, we performed a random 80%/10%/10% training/validation/test split of the in-domain data set, and early stopping was evoked based on validation error. We present the mean absolute error (MAE) of the test split as the predictive performance of interpolation. As shown in Figure S6, we obtain MAEs of 0.057 eV on on-top OH* and 0.064 eV on fcc O* for the test split, where on-top OH* and fcc O* are jointly trained but separately visualized. It is noteworthy that state-of-the-art GNN models^11,22,51,52^ and conventional ML methods^53−55^ typically show MAEs in range of 0.1–0.2 eV for adsorption enthalpy predictions. Our results indicate an excellent interpolation performance.

A more important aspect of this work is to assess the predictive performance of the GNN model for extrapolation tasks, where compositions are dissimilar to those in the training data set. This is because the GNN model will be used to predict a larger HEA design space. To this end, we consider two out-of-domain data sets: (1) Composition-diversity data set: An unknown 5-element space, RuRhPdIrPt, with 120 uniformly sampled compositions. (2) Component-diversity data set: 100 different unknown 5-element spaces, each with four randomly sampled compositions (see Section S1 of the Supporting Information for further details on the out-of-domain data set). Figure 2b–e shows the resulting parity plots of DFT-calculated versus ML-predicted adsorption enthalpies on these two data sets. The predictive performance is excellent, with a MAE of less than 0.1 eV for all predictions. This reflects the practical utility of the model for catalyst discovery. The composition-diversity task (Figure 2b,c) is generally easier than the component-diversity task (Figure 2d,e). This is likely due to the fact that the composition-diversity data set contains a less diverse set of active sites, leading to narrower energy spans of ∼2.5 eV compared to ∼3.5 eV for the component-diversity data set. Furthermore, for the predictions of fcc hollow O* adsorption enthalpies (Figure 2c,e), we obtained MAEs of 0.051 and 0.094 eV, while of 0.039 and 0.074 eV for their on-top OH* counterpart (Figure 2b,d). The slightly higher error for the O* against OH* predictions can be attributed to the more complex local environment of the fcc hollow site.

As for the downstream step, the computationally inexpensive GNN model described above was then used to predict the adsorption enthalpies of all active sites in sufficiently large supercells, e.g., a 100 × 100 supercell containing on the order of 10^5^ active sites, within seconds. The resulting gross adsorption enthalpy distribution is shown as a gray outline in Figure 2a. The current density model subsequently enforces heuristic surface coadsorption rules that are used to obtain the net adsorption enthalpy distribution (see color-filled bars in Figure 2a) and average current density. This model is based on a recent development^11^ (see also the software repository^49^) and has demonstrated good agreement with experimental measurements in previous studies,^9,56^ thus serving as a practically useful target for catalyst design. Although it has predominantly been used for predicting 5-element HEAs, it is also applicable beyond the 5-element HEA space. As an extension, in this work, we will use it together with the extrapolative GNN model to discover higher-dimensional HEA electrocatalysts.

### Biobjective Optimization

Having established a robust ML-based model that can predict catalytic activity for HEAs with arbitrary composition, we first take catalytic activity as our primary objective and, as a second dimension, use cost-effectiveness as a simple but important and commercially valuable objective. Because, in experiment, the fabrication of thin-film HEA libraries is typically carried out using magnetron sputter deposition in a high-throughput manner,^7−9^ cost-effectiveness is directly related to the consumption of each amount of metal species in metallic targets. Therefore, we simply represent the cost-effectiveness as an average cost of the constituent metal species with reference to Pt, i.e., *T*_cost_ = ∑_*i* = 1_^*n*^*c*_*i*_*X*_*i*_^Pt^, where *c*_*i*_ is the individual molar fraction and *X*_*i*_^Pt^ is the relative cost of each metal species as of January 2023.^57^

**Figure 3:**
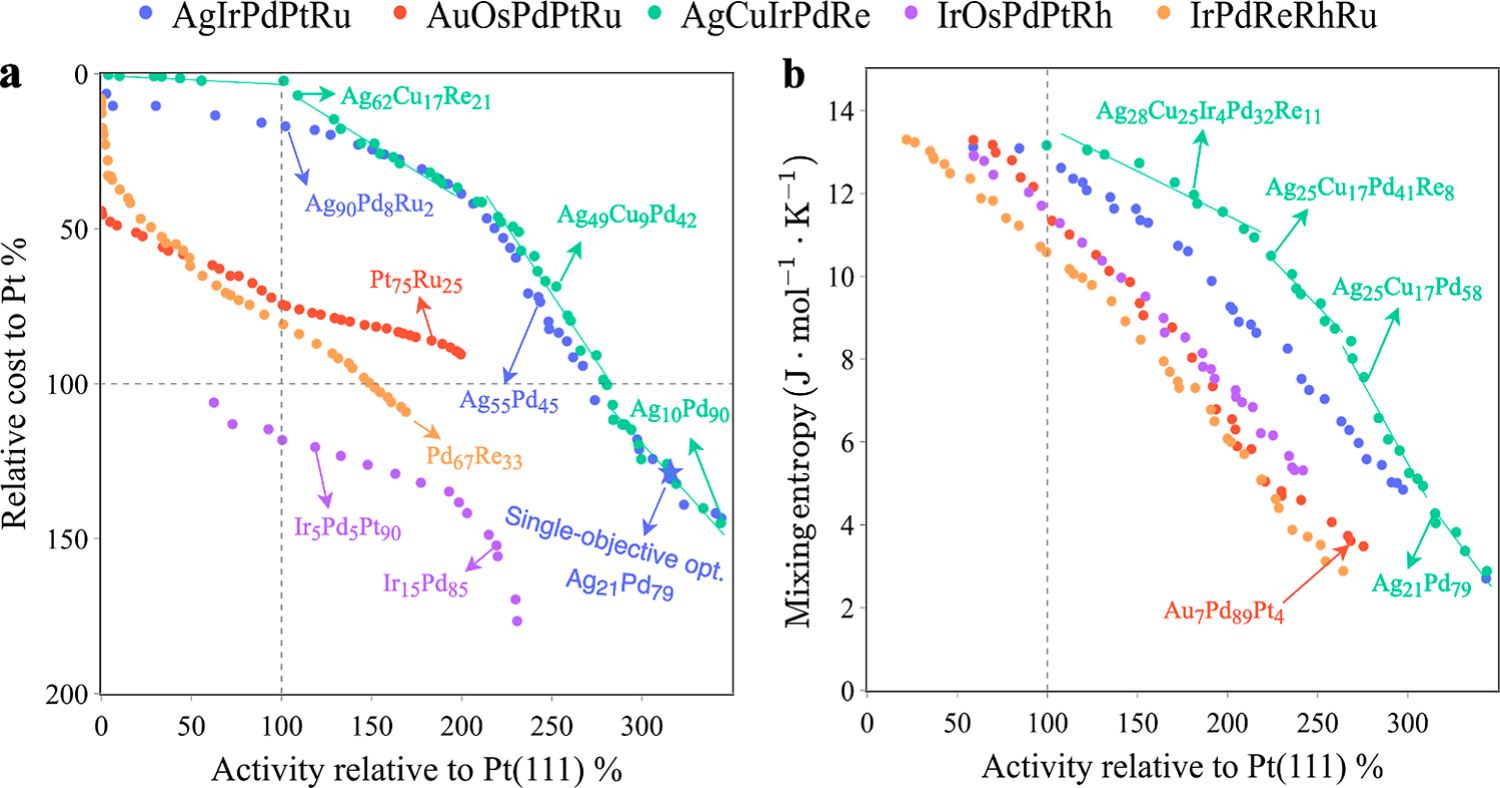
Learned Pareto fronts for five different 5-element HEA spaces obtained using biobjective BO for (a) catalytic activity and relative cost and (b) catalytic activity and mixing entropy. The blue star in (a) represents the result of single-objective BO on the AgIrPdPtRu HEA space. Linear fitting is used to emphasize subclusters on the Pareto fronts, where the corresponding Pareto-optimal set changes smoothly (see text). Gray dashed lines indicate the catalytic activity of Pt(111) and the relative cost of Pt, so that the top right quadrant of the figure contains materials that are both cheaper and more active than Pt.

For ease of illustration, we choose five different 5-element HEA spaces: AgIrPdPtRu, AuOsPdPtRu, AgCuIrPdRe, IrOsPdPtRh, and IrPdReRhRu, where the first two have been synthesized and studied in recent publications.^9,11,21,38^Figure 3a shows the resulting Pareto front of biobjective BO for catalytic activity and relative cost. The Pareto front is defined as the set of nondominated solutions that provide a suitable compromise between all objectives.^41,58^ It is intriguing that, in general, activity and relative cost are conflicting properties, and different 5-element compositions exhibit varied relationships between these two objectives. Specifically, AgIrPdPtRu and AgCuIrPdRe exhibit a broader Pareto front compared to the other three variants, which can be quantified via so-called hypervolume indicators, measuring the volume of the objective space dominated by a given Pareto front (see Figure S9a).

Both AgIrPdPtRu and AgCuIrPdRe are thus promising composition spaces yielding both cheap and high-performing catalysts for the ORR. Furthermore, AgCuIrPdRe shows a slightly higher hypervolume indicator than AgIrPdPtRu. We can attribute this to the presence of Cu in AgCuIrPdRe leading to a lower price and improved activity that can be observed both in a higher-activity region, e.g., Ag_49_Cu_9_Pd_42_ vs Ag_55_Pd_45_ and low-cost region, e.g., Ag_62_Cu_17_Re_21_ vs Ag_90_Pd_8_Ru_2_. It is worth noting that many identified Pareto-optimal solutions contain substantial amounts of Pd and Ag. Nevertheless, the Pareto-optimal solutions exhibit significant compositional differences in terms of the Ag/Pd ratio. This can be seen for Ag_90_Pd_8_Ru_2_ and Ag_21_Pd_79_, both shown in Figure 3a. These candidates are not simply variations drawn from the same optimum nor is the former merely a dilution of the latter. In fact, the resulting Pareto front can be interpreted in terms of subclusters. Particularly, in Figure 3a, we can identify AgPd, AgCuPd, AgCuPdRe, and AgCuRe/CuRe/AgCu subclusters, from right to left on the Pareto front of AgCuIrPdRe. The gradual introduction of Cu and Re along with the reduction of Pt in the catalysts gives rise to not only reduced relative cost but also reduced catalytic activity. Each subcluster in the performance space can be linearly fitted (illustrated by the solid green line in Figure 3) and corresponds to a subcluster in the design space where compositions change smoothly in this local region, ensuring a continuous Pareto front.

It is interesting to compare these results with single-objective BO as the hitherto prevalent approach for optimizing HEA compositions. We therefore follow the recently developed single-objective BO^11,21^ (see also the software repository^49^) for AgIrPdPtRu that is solely focused on catalytic activity (indicated by the blue star in Figure 3a). This method successfully identifies a candidate with exceptionally high catalytic activity. However, as seen from Figure 3a, this candidate might not be desirable in terms of cost considerations. In contrast, the biobjective approach not only encompasses the solutions obtained through single-objective optimization but also provides a range of candidates that are equally valid, considering both lower cost and still relatively high activity. Specifically, there are, for example, several candidates with an activity twice as high as the one of Pt, while simultaneously achieving a cost reduction of around 50% with respect to this reference material.

Notably, this extended insight of the biobjective BO comes along without compromising the well-known data efficiency of single-objective BO. Twenty to 50 evaluations are required to reach 95% of the maximal hypervolume indicator (see Figures S9a, S10 and Tables S2, S3), which compares perfectly with the ca. 50 evaluations needed to find the global optimum of the 5-element space in single-objective BO.^21,23^ This high data efficiency can be ascribed to dedicated implementations in our diversity-guided multiobjective BO approach that include a first-order approximation in the Pareto-discovery solver, the diversity-guided batch selection scheme, and a kernel-based surrogate model, i.e., Gaussian process regression (GPR). The results concerning the number of experiments required for single-objective and multiobjective BO are presented in Tables S2–S5, and a discussion of data efficiency is provided in Section S4.3 of the Supporting Information.

Besides catalytic activity and cost-effectiveness, another essential objective in the development of HEAs is mixing entropy. A low mixing entropy typically means that the system will tend to form intermetallics or multiple solid solutions, making it challenging to achieve a single solid solution (HEA) in synthesis.^59,60^ Furthermore, such a HEA is likely less stable as a catalyst under reactive conditions since it would have a tendency to evolve toward the thermodynamically preferred (e.g., intermetallic) state. It is noteworthy that the omission of the entropic effect in single-objective optimization often leads to the selection of top-ranking HEA candidates that are, in fact, mere binary alloys. To address this, we performed a biobjective optimization on catalytic activity and mixing entropy for the same set of five 5-element HEA spaces as before and utilizing an ideal mixing entropy rule: Δ*S*_mix_ = −*R*∑_*i* = 1_^*n*^*c*_*i*_ ln *c*_*i*_, where *R* is the ideal gas constant and *c*_*i*_ is the molar fraction of element *i*. We also tested the correlated mixing entropy indicator reported in refs (61 and 62,) which is an empirical model taking into account the contributions of atomic size and bond mismatch to the entropy (see Section S6 in the Supporting Information). Highly comparable findings are obtained with both methods.

As demonstrated in Figure 3b, mixing entropy and activity are, in general, conflicting objectives. A distinct interplay can be observed between activity/mixing entropy against activity/cost-effectiveness across the five HEA spaces. However, AgIrPdPtRu and AgCuIrPdRe are still more promising than others in terms of the area covered by the Pareto front. Further analysis of the AgCuIrPdRe front reveals that there is a subcluster that contains HEAs composed of all five elements in the lower-activity region, whereas AgCuRe/CuRe/AgCu subclusters appear in the corresponding region of the activity/cost-effectiveness performance space (see Figure 3a). In addition, while AgPd binary alloys demonstrate remarkably high catalytic activity, our analysis reveals a range of ternary and quaternary alloys with equally appealing properties, achieving 240–280% catalytic activity [relative to Pt(111)] coupled with a high 8–10 J·mol^–1^·K^–1^ mixing entropy.

**Figure 4:**
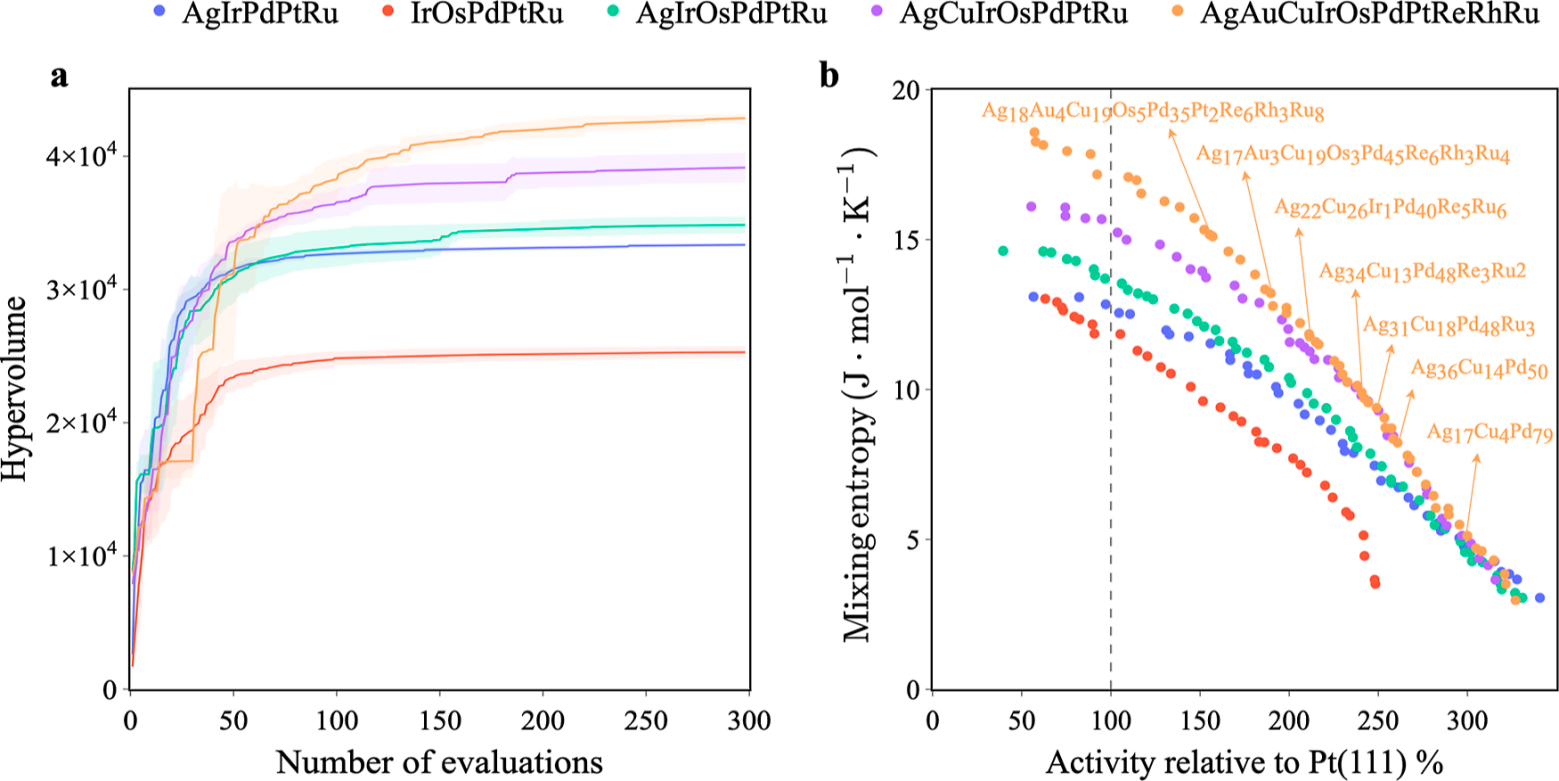
Learned Pareto fronts for five 5- to 10-element HEA spaces using biobjective BO for catalytic activity and mixing entropy. (a) Hypervolume indicator as a function of the number of evaluations, where the curve is averaged over five different seeds, and the variance is represented as a shaded region. (b) Resulting Pareto front with highlighted Pareto-optimal solutions. Gray dashed lines indicate the catalytic activity of Pt(111).

Next, we increase the dimensionality of the design space from 5D to 10D and perform a biobjective BO on catalytic activity and mixing entropy. Two overlapping 5-element spaces, namely AgIrPdPtRu and IrOsPdPtRu, as well as 6D, 7D, and 10D spaces, are considered where the higher-dimensional spaces are chosen to encompass the lower-dimensional ones. We present hypervolume indicators against the number of evaluations in Figure 4a. It is not surprising that higher-dimensional spaces require a larger number of evaluations to converge: 5-, 6-, 7-, and 10-element HEA spaces used around 50, 100, 110, and 140 evaluations to reach 95% of the maximum hypervolume, respectively. Compared to the orders of magnitude increase in the size of the design space, this increase in the number of required evaluations is nevertheless quite modest and attests to the high data efficiency of the approach. Importantly, our biobjective framework exhibits self-consistent results in that the Pareto front of the low-dimensional space is covered by its superset higher-dimensional space (see Figure 4b), in agreement with their hypervolume indicators (see Figure 4a). By increasing the dimensionality, we find that the fraction of Pd can be gradually decreased, and we are able to identify a number of senary, septenary, and octonary alloys exhibiting enhanced catalytic activity of 190–230% relative to Pt and associated with high mixing entropies of 11–13 J·mol^–1^·K^–1^. Note that these alloys are not accessible in low-dimensional spaces, and they can neither be obtained through biased component sampling anymore as there are too many combinations to consider.

Importantly, our findings on biobjective optimization are in good agreement with experiments. Prior studies have highlighted the favorable electrocatalytic ORR activities of identified binary alloys such as AgPd^63,64^ and IrPt^65,66^ (see Figure 3a), as well as ternary alloys like AuPdPt^67,68^ and AgCuPd^69^ (see Figure 3b). In particular, the optimal compositions were determined to be around Ag_10_Pd_90_^64^ and Ir_15_Pt_85_,^65^ which aligns well with our results. For systems with more elements, such as the quaternary (AgPdPtRu)^56^ and quinary (AgIrPdPtRu)^9^ alloys, our method is inherently capable of predicting the experimental outcomes. This is because it uses the same current density modeling technique as in refs (9) and (56), and because the AgIrPdPtRu data set used therein is a subset of our training data. It should be further noted that Batchelor et al.^9^ restricted the fraction of constituting five elements to a narrow composition range, e.g., Ag_1–9_Ir_8–18_Pd_17–49_Pt_12–33_Ru_17–52_, and carried out theoretical predictions using a grid search. Our method can identify these quinary alloys more efficiently, suggesting Pareto-optimal candidates that are discovered in a wider composition range.

### Triobjective Optimization

**Figure 5:**
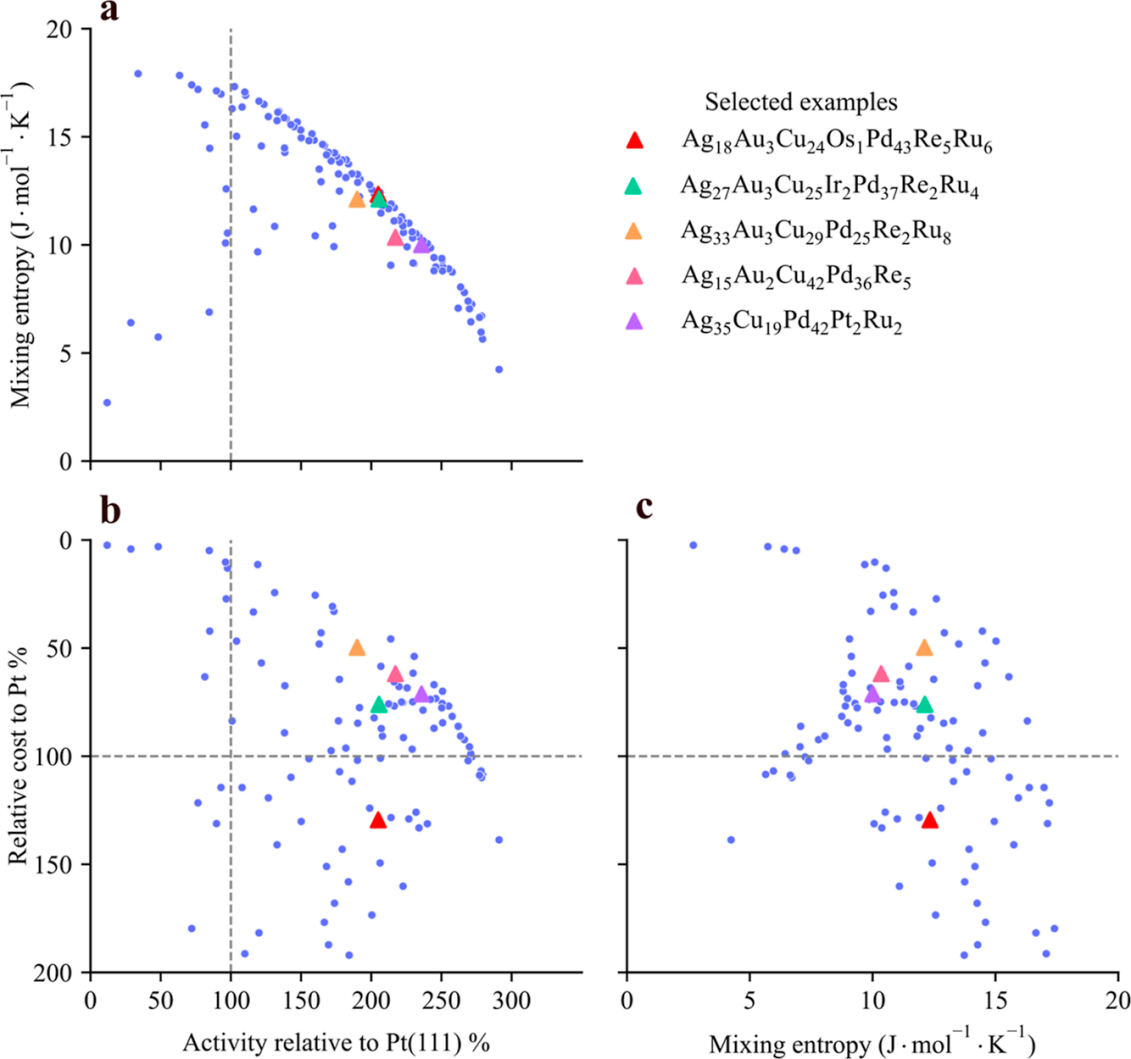
Learned Pareto fronts for a 10-element HEA space (AgAuCuIrOsPdPtReRhRu) using triobjective BO for catalytic activity, cost-effectiveness, and mixing entropy. The 3D Pareto fronts are projected to three pairwise 2D subplots. Selected Pareto-optimal solutions are highlighted as triangles.

Up to this point, we have demonstrated the usefulness of our general multiobjective BO framework for biobjective optimization and its advantages over single-objective optimization. Next, we combine all complexities, namely, three objectives and a 10-dimensional design space, to conduct a comprehensive triobjective optimization. To illustrate the results, we employ a pair plot (see Figure 5) to visualize the learned 3-dimensional Pareto fronts projected onto each pairwise combination of two dimensions for the 10-element HEA space (AgAuCuIrOsPdPtReRhRu). It is fascinating to observe that the distribution of Pareto fronts in the projected two dimensions differs significantly from those obtained through biobjective optimization (e.g., Figure 4b). Many Pareto-optimal solutions are situated inside the frontier. While these solutions may perform worse in the projected 2D space, they succeed in the third objective.

Similar to the results of the 10-element HEA in Figure 4b, Figure 5a showcases numerous candidates exhibiting an appealing compromise between activity and mixing entropy. The Pareto-optimal solutions found in these regions may not outperform standard Pt in every objective though. For example, candidates such as Ag_18_Au_3_Cu_24_Os_1_Pd_43_Re_5_Ru_6_ (red triangle) exhibit a catalytic activity of 205% and a mixing entropy of 12.3 J·mol^–1^·K^–1^, but come with a relatively higher cost (130%). However, a spectrum of intriguing Pareto-optimal solutions indeed demonstrates satisfactory performance across all objectives. Examples include 5-element Ag_35_Cu_19_Pd_42_Pt_2_Ru_2_, Ag_15_Au_2_Cu_42_Pd_36_Re_5_, 6-element Ag_33_Au_3_Cu_29_Pd_25_Re_2_Ru_8_, and 7-element Ag_27_Au_3_Cu_25_Ir_2_Pd_37_Re_2_Ru_4_, which exhibit activity/entropy/cost in the range of 190–235%, 10–12 J·mol^–1^·K^–1^, and 50–75%, respectively.

Furthermore, despite exhibiting similar performance across all objectives, an analysis of the compositions in these four examples reveals that they do not belong to the same local minima in the design space. For instance, the proportions of Ag and Cu differ significantly between Ag_35_Cu_19_Pd_42_Pt_2_Ru_2_ and Ag_15_Au_2_Cu_42_Pd_36_Re_5_, while the fractions of Cu, Pd, and Ru vary notably between Ag_33_Au_3_Cu_29_Pd_25_Re_2_Ru_8_ and Ag_35_Cu_19_Pd_42_Pt_2_Ru_2_. This underscores the advantage of our multiobjective optimization workflow, which is capable of exploring different regions of the compositional landscape. In general, the candidates resulting from triobjective optimization are more promising in terms of cost-effectiveness. Such candidates are not easily discovered through biobjective optimization. A full list of discovered Pareto-optimal candidates can be found in Section S7 of Supporting Information.

We have also compared the Pareto fronts obtained from the 10-element HEA space with those from a selected 5-element HEA space (see Figure S11). A broader frontier is evident across all three pairwise combinations of two dimensions. These findings suggest that our multiobjective BO framework explores more promising regions in the 10D space and manages to surpass the compromise among three objectives in the 5D space. It is noteworthy, however, that there is a smaller proportion of Pareto-optimal solutions in extreme scenarios, such as very low (less than 30%) and high (greater than 300%) catalytic activity regions. This phenomenon can be attributed to the limited exploration of extreme scenarios, a known issue referred to as the “long-term local minima problem”.^70^ The algorithm may become trapped in local minima for extended periods, resulting in insufficient exploration of these extreme scenarios. Nonetheless, it is important to mention that these extreme scenarios often involve less favorable materials, such as binary alloys with low cost-effectiveness and mixing entropy, which make them less relevant to our research objectives.

After presenting the notable findings in biobjective and triobjective optimizations, we now turn our attention to discussing the remarkable ability of our multiobjective BO framework to explore high-dimensional HEA design spaces. First, the batch selection strategy employed in our diversity-guided BO methods takes into account the diversity in both design and performance spaces. This strategy facilitates the favorable selection of dissimilar compositions and drives the discovery of a broader Pareto front in the performance space. The inclusion of these diverse compositions further enhances the predictive abilities of the GPR surrogate model, enabling a reliable exploration of different regions in this vast design space.

Second, the diversity-guided multiobjective BO method incorporates various design choices which allow the search to escape from local minima, such as hypercubic sampling and stochastic sampling in the Pareto-discovery solver. Third, our GNN model with superior extrapolative performance allows for increasing the dimensionality of the design space. The key to predicting the catalytic activity of arbitrary HEAs is to address the diverse local environment of the active sites. Although the training set includes only 5-element HEAs, the use of uniformly sampled compositions ensures a significantly diverse set of slabs and consequentially of active sites. In addition, it is important to include some chemical similarity in the training set or embeddings into the extrapolation tasks. For instance, this work includes all targeted elements in the training set, which is consistent with a previous study that has shown that even minimal information about an element can significantly improve extrapolation performance.^53^

We believe that ML surrogate model-driven multiobjective optimization will revolutionize the search for more realistic and multifunctional materials. Our multiobjective BO framework represents a versatile approach in which we have demonstrated that the utilization of the GNN model can efficiently and reliably handle the complexity in HEAs. In essence, one can leverage various ML surrogate models to predict multiple intricate target properties of interest. The speed and efficiency of these ML surrogate models make them highly suitable to be integrated with multiobjective optimization algorithms, thus enabling the evaluation of a large number of potential candidates and quickly locating promising regions in the design space.

The multiobjective BO framework is also potentially very useful in driving high-throughput experiments. The batch selection strategy allows the conduction of many experimental tests in parallel, which is valuable in nontrivial experiments that can take days or months to complete. While our framework is data-efficient by design through an effective selection strategy and Pareto-discovery solver, there is room for further improvement as it may not be necessary to discover the entire continuous Pareto front for experiments. We believe that focusing on identifying a few representative points within subclusters and subsequently interpolating within those subclusters can offer valuable efficiency, thus further reducing the number of experiments required. We leave this direction for further development and exploration.

The main remaining limitation of our multiobjective BO framework is the simplified assumption of our surface model. Although the fcc(111) surface with a random arrangement of atoms is a good first approximation of HEA electrocatalyst surfaces that has been successfully applied in many theoretical studies,^11,14−16,19−22^ recent experiments have observed the formation of multiphases,^8^ which suggests that these assumptions may not be completely accurate for describing the true catalyst surface. At the present, this is a frontier area of HEA research. We anticipate that the development of a better ML model for phase prediction^71^ and further experimental characterization can help us improve such surface models.

## Conclusions

In summary, we have developed a data-efficient multiobjective BO framework tailored for the discovery of HEA electrocatalysts for ORR. This framework advances beyond the state-of-the-art by tackling higher dimensional HEA spaces and expands beyond the prevalent single-objective BO approaches by enabling the discovery of Pareto-optimal catalysts. This is achieved by integrating an extrapolative GNN model with a variety of design strategies inherent to multiobjective BO. This allows for effective exploration in a targeted 10-element HEA space, even when exclusively training on 5-element HEAs. By concurrently targeting three key objectives (catalytic activity, cost-effectiveness, and entropic stabilization), our method has effectively identified a diverse range of promising HEA electrocatalysts. These materials achieve a balance among all objectives, which are unattainable with single-objective BO. The identified optimal binary to quinary HEAs are supported by previous experimental results, whereas those with more elements are awaiting experimental validation. We underscore that our data-efficient multiobjective BO approach is versatile and applicable to both theoretical screening and high-throughput experiments, accommodating various targeted objectives. The reduced number of evaluations required signifies an encouraging advancement, indicating that optimizing HEA compositions within vast compositional spaces is experimentally feasible in a laboratory setting.

## Data Availability

The DFT data set that supports the findings of this study is available in the GitHub repository at https://github.com/Wenbintum/MOBO_HEAs_data. The source code necessary to reproduce all experiments is available on Google Drive at: https://drive.google.com/drive/folders/128fvqLjgNLpwhFpXtyv9p3C1bymGpkCH?usp=share_link. A brief introduction and tutorial for the source code can be found on Google Colab at: https://colab.research.google.com/drive/1yj8Oulglmk-rfRceV41_c-3_9Arl8Rxf?usp=share_link.

## Supporting Information Available

Additional details on the DFT data set, DFT computational details, the GNN model, the multiobjective BO method, current density modeling, correlated mixing entropy, and a full list of Pareto-optimal solutions (PDF)
