# Supplementary material for: Discovering High Entropy Alloy Electrocatalysts in Vast Composition Spaces with Multiobjective Optimization
Source: J Am Chem Soc. 2024 Mar 11;146(11):7698–707. doi: 10.1021/jacs.3c14486 (PMC10958507; doi:10.1021/jacs.3c14486)
Supplement: Supplementary file 1 — ja3c14486_si_001.pdf [file ja3c14486_si_001.pdf]

# Supporting Information: Discovering high entropy alloy electrocatalysts in vast composition spaces with multi-objective optimization

Wenbin Xu,[†,‡] Elias Diesen,[†] Tianwei He,[¶] Karsten Reuter,[†] and Johannes T. Margraf[*,†,§]

†*Fritz-Haber-Institut der Max-Planck-Gesellschaft, Berlin, D-14195, Germany*

‡*Lawrence Berkeley National Laboratory, Berkeley, CA 94720, USA*

¶*Yunnan Key Laboratory for Micro/Nano Materials & Technology, National Center for International Research on Photoelectric and Energy Materials, School of Materials and Energy, Yunnan University, Kunming, 650091, China*

§*University of Bayreuth, Bayreuth, D-95447, Germany*

E-mail: johannes.margraf@uni-bayreuth.de

# S1 DFT dataset

Two distinct DFT datasets have been established in this work: the in-domain dataset and the out-of-domain dataset. The in-domain dataset serves as the training set for the ML regression model used in predicting adsorption enthalpies. It contains 19,955 data points and comprises three 5-element HEA subsets: AgIrPdPtRu, AuOsPdPtRu, and CuPtReRhRu, covering a total of ten elements. The AgIrPdPtRu subset is taken from reference,[1] while the last two subsets were constructed using DFT calculations with the same settings and post-processing steps to ensure data consistency. The data preprocessing is intended to filter out relaxed structures where the slabs have converged in a rearranged state or where the adsorbate has moved to a different site. It is noteworthy that the existence and viability of the AgIrPdPtRu and AuOsPdPtRu HEA catalysts have been confirmed through recent experimental studies,[2,3] and Cu-based catalysts have shown promising potential.[4,5] To be more specific, we considered fcc(111) surfaces with randomly distributed surface atoms and OH* and O* adsorbed in on-top and fcc hollow sites. To generate diverse slab compositions, compositions were drawn from a Dirichlet distribution with uniform density within the 5-dimensional simplex space. In this spanned hyperspace, samples located at the edges and corners exhibit less diverse active site motifs, which are generally easier to predict compared to those at the center. The large diversity resulting from this sampling method can aid the ML model in making predictions throughout the spanned hyperspace. The atom identities were then picked from that set of probabilities. Next, on-top OH* and fcc hollow O* adsorption motifs were randomly sampled nine times for each unique slab. After carrying out the necessary postprocessing steps, we obtained an in-domain dataset with sizes of 5,039, 7,461, and 7,455 for AgIrPdPtRu, AuOsPdPtRu, and CuPtReRhRu, respectively. This in-domain dataset provides a comprehensive and diverse representation of the surface configurations and concomitant active sites.

The out-of-domain dataset contains 4,020 data points and comprises two subsets: composition-diversity and component-diversity subsets for the purpose of testing extrapolative perfor-

mance. The composition-diversity subset focuses on an unknown 5-element RuRhPdIrPt. To generate this subset, we sampled 120 slabs with different compositions using the Dirichlet distribution method described above. For each unique slab composition, the on-top OH* and fcc hollow O* adsorption motifs were randomly sampled nine times individually. The component-diversity subset contains 100 different 5-element combinations randomly drawn from the 10-element library. For each 5-element combination, we randomly sampled four compositions, and for each composition, three different on-top OH* and fcc hollow O* adsorption motifs were randomly selected. By conducting the same postprocessing step as the in-domain dataset and previous studies,[1] we end up with an out-of-domain dataset with sizes of 2,138 and 1,882 for composition-diversity and component-diversity subsets, respectively. The adsorption enthalpy distributions for the in-domain and out-of-domain datasets are shown in Figs. S1 and S2, respectively. Additional information on the DFT calculations is provided in Section S2.

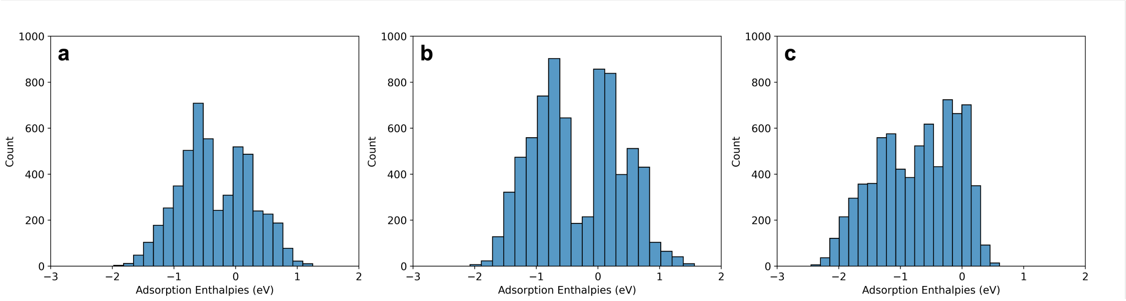


Figure S1: Distribution of adsorption enthalpies of in-domain dataset for (a) AgIrPdPtRu (b) AuOsPdPtRu, and (c) CuPtReRhRu subset.

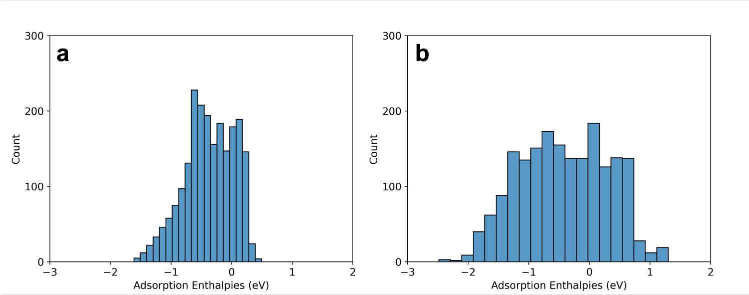


Figure S2: Distribution of adsorption enthalpies of out-of-domain dataset for (a) composition-diversity and (b) component-diversity subset.

# S2 DFT computational details.

The DFT calculations of in-domain and out-of-domain datasets were performed using the following settings, which are entirely consistent with previous studies.[1] The GPAW code[6,7] was used with a plane-wave basis set and the revised Perdew-Burke-Ernzerhof (RPBE) exchange-correlation functional.[8] The HEA surfaces were modeled using the fcc(111) slab with a randomly distributed atomic arrangement to represent random solid solutions. We used a 3 × 3 atom-size surface cell and five atomic layers. The larger surface size was meant to capture the intricate local electronic environment,[1,9] and the deeper layers managed to account for long-range and directional ligand effects in HEAs.[10] The bottom two layers were kept fixed in their bulk-truncated positions, while the top layers and adsorbates were relaxed until the maximum force on each atom was below at least 0.1 eV/Å. All DFT calculations were conducted as periodic slab calculations, with a vacuum region of 10.0 Å above and below the slab. A 4 × 4 k-point grid and a plane waves energy cutoff of 400 eV were employed. The initial surface structure setup and DFT geometry optimization were carried out using ASE[11] and CHEAT packages.[1] Specifically, on-top OH* and fcc hollow O* were positioned above the surface at distances of 2.0 Å and 1.3 Å, respectively. After geometry optimization, the adsorption enthalpies were calculated as $\Delta E_{\mathrm{ads}} = \Delta E_{\mathrm{HEA+ads}} - \Delta E_{\mathrm{HEA}} - \Delta E_{\mathrm{Pt(111)+ads}} + \Delta E_{\mathrm{Pt(111)}}$, where $\Delta E_{\mathrm{HEA+ads}}$ and $\Delta E_{\mathrm{HEA}}$ are the calculated total energy of the HEA slab with and without adsorbate (i.e., O* or OH*) and $\Delta E_{\mathrm{Pt(111)+ads}}$ and $\Delta E_{\mathrm{Pt(111)}}$ donate the calculated total energy of the Pt(111) reference slab with and with without adsorbate.

# S3 Additional details on the Graph Neural Network (GNN) model

Graph representation is a versatile approach for representing various molecular systems, including isolated molecules, crystal structures, and surface-adsorbate systems. Incorporating graph representation into kernel method or graph neural networks has led to state-of-the-art accuracy of $0.1 - 0.2$ eV for adsorption enthalpy prediction.[1,12–15] In this study, we utilize a variant of gated graph convolutional networks[16] that has recently been developed for predicting 5-element HEA catalysts,[1] in which a long-range node feature related to direction-dependent effects in the third layer of the surface slab, is included (see Fig. S3 for the GNN architecture). The GNN model relies on the local environment (only connectivity) of the active site, where we consider graph representations that encompass up to second-rank neighbors, equivalent to three node distances to the binding atom of the adsorbate. In this way, the GNN model is size-extensive, enabling predictions for sufficiently large surface cells. The graph edges between two atoms were determined based on their covalent radii with a skin of 0.3 Å. The node attribute used consists of a 14-dimensional feature vector. This vector includes a 12-dimensional element onehot encoding, representing the ten different metal species and O and H in the adsorbates. Additionally, it includes a one-dimensional zone feature and a one-dimensional long-range feature. The long-range feature is determined based on whether the third layer exhibits a direction-dependent effect through the second zone atom to the binding atom. More details about the long-range feature can be found in the Fig. S4. We also tested the use of a learnable atom embedding, which is initialized based on the atom's identity.[17] However, the predictive performance of this approach was found to be inferior compared to the inclusion of the long-range feature within the current GNN architecture. This can be attributed to the fact that while graph representation already includes connectivity information about how atoms bond to other atoms, the long-range feature provides additional directional information that points out the underlying atoms for

the adsorption. Therefore, we stick with the generated 14-dimensional node features.

The GNN model was implemented using the PyTorch framework, Adam optimizer and MAE loss function. We use a random 80%/10%/10% training/validation/test split of the in-domain dataset. During the training process, an early stopping strategy was used to prevent overfitting where we monitored the validation error by calculating the rolling mean over a window of 10 epochs, which is compared to that of all prior epochs. If the rolling mean validation error did not decrease by at least 1% compared to the previous lowest error within 100 epochs, early stopping was triggered. The learning rate was set to 0.001. To optimize the hyperparameters of the GNN model, we conducted a systematic grid search on various parameters including dimensionality of convolutional layers, number of convolutional layers, number of hidden layers, and batch size. We evaluated the performance of each combination of hyperparameters using the MAE of validation. The results of the grid search, along with the associated validation MAE values, are presented in a parallel coordinates plot (see Fig. S5). Based on the grid search results, we identified a set of optimal hyperparameters that are 22, 4, 0, 64 for dimensionality of convolutional layers, number of convolutional layers, number of hidden layers, batch size, respectively, yielding robust predictions. We then reported the MAE on test split as the predictive performance of the in-domain prediction (see Fig. S6).

For the extrapolation tasks, using the identified optimal hyperparameters, we performed a random 90%/10% split of the in-domain dataset for training and validation and used same early stopping strategy described above. We then assessed the model's performance on the composition-diversity and component-diversity datasets, which serve as the out-of-domain predictions (see Figs. 2b-e in the main text). The resulting pretrained GNN model will be used to infer predictions in multi-objective optimization.

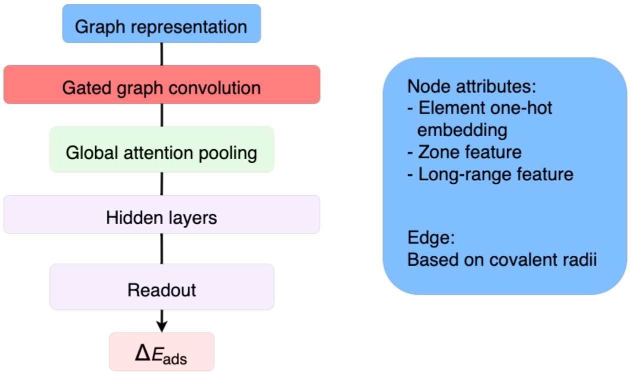


Figure S3: Schematic illustration of the GNN architecture. The elemental species are encoded as one-hot vectors with a size of 12 (i.e., 10 metal species, hydrogen and oxygen). "Zone feature" refers to the sequence number of zone that an atom presents (e.g., the zone feature of an adsorbate atom is 0, that of the active site atom is 1, and that of the first nearest neighbor is 2, etc). The long-range feature is described in Fig. S4. The graph edges between two atoms were determined based on their covalent radii with a skin of 0.3 Å.

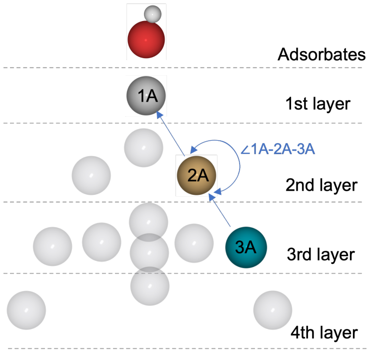


Figure S4: Illustration of the long-range feature used in node attributes of the GNN model. The long-range feature is determined based on whether the third layer exhibits a direction-dependent effect through the second zone atom to the binding atom, i.e., the angle of $\angle$ 1A-2A-3A $> 150°$. Redrawn based on Fig.4 in Ref. 10.

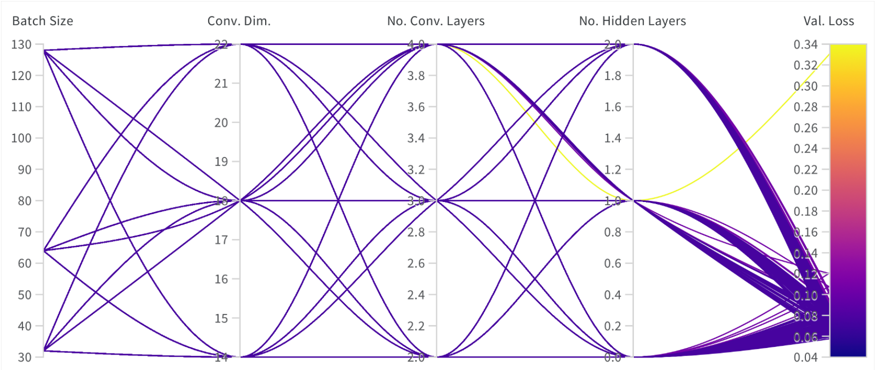


Figure S5: Parallel coordinates plot of the hyperparameter search of the GNN model. A grid search was performed on the dimensionality of convolutional layers, number of convolutional layers, number of hidden layers, batch size, and learning rate. Validation loss is in eV.

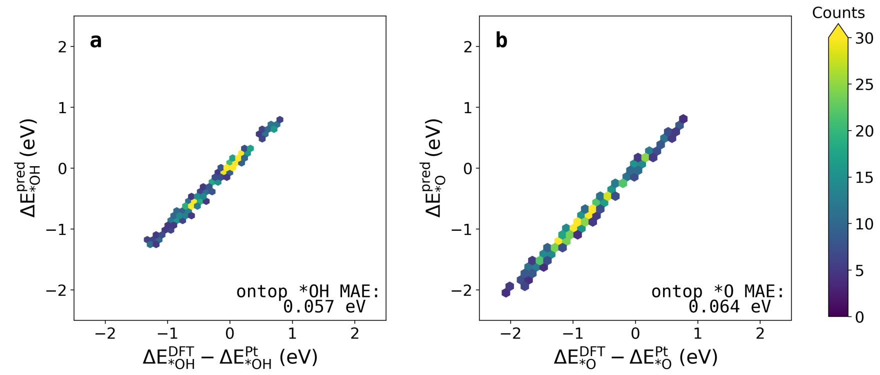


Figure S6: Parity hexbin plot of DFT-calculated vs. ML-predicted adsorption enthalpies for in-domain predictions of (a) on-top OH*, (b) fcc O* on test split. The colorbar denotes the number of points in each hexbin.

# S4 Additional details on the diversity-guided multi-objective Bayesian optimizaiton (BO)

Our customized multi-objective BO framework is built upon a recently developed diversity-guided approach.[18] In general, multi-objective optimization methods consist of four key elements, including the surrogate model, acquisition function, solver, and selection strategy. Specifically, in this work we utilize GPR with a radial basis function kernel, the identity function, the Pareto-discovery solver,[19] and a diversity-guided selection strategy, respectively. For more details on each of these key elements, a thorough comparison to single-objective optimization methods used in previous studies,[1,20] and hyperparameters used, please refer to sections S4.1 and S4.2.

Furthermore, we mindfully customized the diversity-guided approach[18] for our HEA application in following ways: 1) For a given number of elements $N$, we are addressing an optimization problem subject to an equality constraint that requires the sum of the fractions of the individual elements to be 1. To efficiently handle this equality constraint problem, we reformulated it to inequality constraints: $\sum_1^{N-1} C_i - 1 < 0$ and $C_n < 0.9$, where the individual fraction is limited to 0 to 0.9. In this way, the number of variables is $N-1$, and the $N^{\text{th}}$ variable is set to be deterministic. Note that the limit for the $N^{\text{th}}$ element $C_N$ are from 0 to 0.9. Since $C_N$ is determined by the equation $C_N = 1 - \sum_1^{N-1} C_i$, its lower limit occurs when the sum of fractions ($\sum_1^{N-1} C_i$) is at its maximum, just below 1, making $C_N$ slightly greater than 0. The upper limit of $C_N$ is directly imposed by the second constraint, $C_n < 0.9$. 2) The original method employed a Latin hypercube sampling in both the initial sampling step and stochastic sampling step of the Pareto-discovery solver. However, it is hard to get valid samples that satisfy the equality constraint, e.g., $\sum_1^{N} C_i = 1$. Instead, we draw variables from a uniform hypercubic sampling and transform them using quantiles of gamma functions followed by normalization, resulting in a Dirichlet-like distribution. This sampling method gives rise to a better discrepancy score than standard Dirichlet sampling,

indicating a better coverage of the design space. 3) The original implementation for computing the gradient vector at the local optimization step in the Pareto-discovery solver can lead to invalid solutions that are out of bounds. We improved it by gradient numerical differentiation respecting bounds.

## S4.1 Multi-objective and single-objective BO Methods

In general, the key components in multi-objective optimization can be classified into the surrogate model, acquisition function, solver, and selection strategy. In Fig. S7, we outlined our customized multi-objective BO method[18,19] in terms of these four components, and we also compared it with a single-objective BO method that was developed recently for discovering 5-element HEA electrocatalysts.[1,20,21]

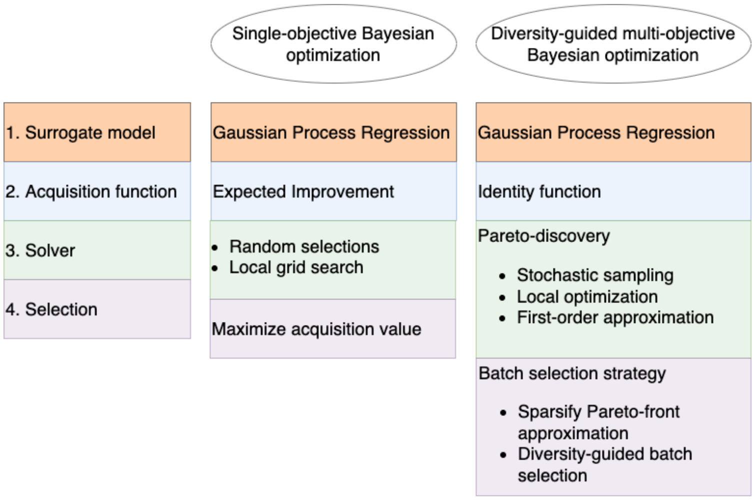


Figure S7: A general outline of multi-objective optimization including surrogate model, acquisition function, solver and selection. The diversity-guided multi-objective BO and single-objective BO used in this work were presented in line with this outline.

The surrogate model serves to approximate an unknown function that maps the design space to the performance space for each objective. In our multi-objective BO method, we utilize Gaussian Process Regression (GPR)[22] with a radial basis function kernel (eq. 1) as the surrogate model. The choice of GPR is motivated by its data efficiency compared to deep neural networks while also providing uncertainty prediction. It is noteworthy that

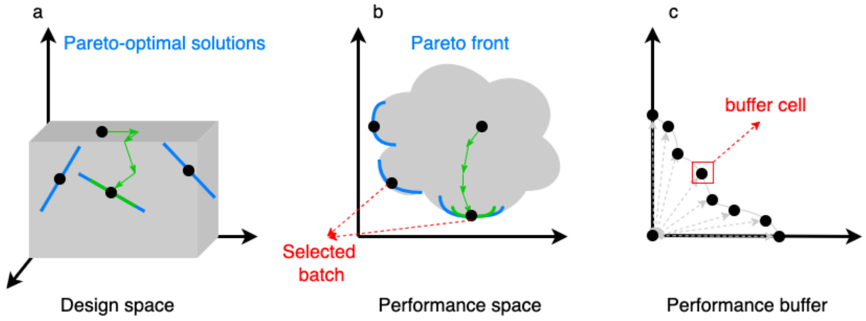


Figure S8: (a-b) Schematic illustration of the Pareto-discovery solver and batch selection strategy. Blue line and blue curve represent Pareto-optimal solutions in the design space and its corresponding Pareto front in the performance space. The green arrow refers to one iteration of Pareto-discovery solver. Batch samples are selected from different subclusters of the Pareto-front using a greedy approach. (c) Performance buffer data structure used to store candidates in the process of multi-objective BO. Each buffer cell includes the points with the minimum distance to the origin that intersects its corresponding ray and also top K candidates within an allowed tolerance of the minimal distance (these hyperparameters were presented in Tab. S1). This buffer gets updated with each iteration of the discovery algorithm. Redrawn from the inserts in Ref. 18.

the number of surrogate models used in multi-objective BO is equivalent to the number of objectives to be optimized. The radial basis function kernel has been successfully applied in many case studies due to its similarity to the Gaussian distribution, leading to smooth and infinitely differentiable functions.

$$K(X, X_*) = c_1 + c_2 \times \exp(-\frac{\|X - X_*\|^2}{2l^2}) \tag{1}$$

The acquisition function is a mathematical technique that provides guidance on how the design space should be explored during optimization. For single-objective BO, the most commonly used acquisition functions include expected improvement,[23] probability of improvement,[24] and upper confidence bound.[25] In the context of multi-objective optimization, the acquisition function is adapted or jointly used to accommodate multiple surrogate models, such as expected hypervolume improvement[26] and predictive entropy search.[27] In this study, we employ an identity function, simply the mean of the GPR posterior. This acquisition function will be incorporated with the Pareto-discovery solver to guide the exploration

of the design space.

Regarding the solver, this work utilizes the Pareto-discovery solver,[19] which aims to discover a continuous Pareto front instead of discrete points, enabling efficient navigation of the compromised landscape. The Pareto-discovery solver operates iteratively and consists of three main steps: stochastic sampling, local optimization, and first-order approximation. Firstly, the stochastic sampling combines newly generated samples from our adapted hypercubic sampling and samples previously found, perturbing them to avoid local minima. Secondly, a local optimization is performed on these perturbed samples, aiming to push each sample towards Pareto-optimal solutions, for which we employ the Sequential Least Squares Programming (SLSQP) method[28] implemented using SciPy,[29] along with gradient numerical differentiation respecting bounds. Next, once a local Pareto-optimal solution is identified, first-order approximation locally expands the Pareto-optimal solutions around this point. This first-order approximation relies on the Karush-Kuhn-Tucker (KKT) conditions,[30] efficiently resulting in a dense set of solutions that approximates a piecewise continuous region of the Pareto front. A complete loop is depicted by a green arrow in Fig. S8, and this loop is applied multiple times to continuously push the samples towards the Pareto-optimal solutions, ultimately resulting in a dense set of approximated Pareto-front shown in the blue curve of Fig. S8b. It should be noted that the Pareto-discovery solver relies on a data structure, namely, the performance buffer shown in Fig. S8c. This buffer is represented as an array discretized using hyperspherical coordinates. Each cell in the buffer stores a list of solutions, including Pareto-relevant points and their associated manifold approximation. Throughout the iterative discovery procedure, the buffer is continuously tracked and updated.

The batch selection procedure begins with the Pareto-front approximation obtained from the Pareto-discover solver. The initial step involves sparsifying the approximated Pareto-optimal solutions, where the idea is to select a single solution from each buffer cell while ensuring that solutions in adjacent buffer cells are close in the design space. This is achieved

by using a graph-cut method that extracts a sparse subset of optimal points grouped into k linear subspaces. The points are grouped based on their values in the performance space and nearness in the design space. Thus, these linear subspaces define diversity regions (as shown by the blue curve in Fig. S8b). Subsequently, the downstream diversity-guided batch selection aims to choose a batch of samples from these regions that are diverse in both the design and performance space, while also maximizing hypervolume improvement (see selected points in Fig. S8b). This is accomplished through a greedy approach. It is remarkable that the selected batch of samples can prevent the optimization process from getting trapped in local minima and can improve the predictive performance of the surrogate model, i.e., GPR, especially when there are significant uncertainties in the initial iterations.

In comparison to the single-objective BO shown in Fig. S7, the main distinction lies in the solver and selection strategy. Specifically, the single-objective BO method employs 1000 randomly selected samples and evaluates their acquisition values. Once a sample with the maximum acquisition value is found, a grid search is conducted to assess samples around that maximum. However, a major issue can arise if the surrogate model is not trained on effective samples, resulting in poor predictive performance. This can lead to incorrect assignment of acquisition values to suggested samples and ultimately getting stuck in local minima. In contrast, multi-objective BO method addresses this problem by incorporating diversity information in both the design and performance space, and various implementations in the Pareto-discovery solver are used to escape from local minima, which significantly alleviates this issue.[18]

## S4.2 Hyperparameters in multi-objective BO

For most of the hyperparameters, we maintained consistency with the previous implementation,[18] as shown in Tab. S1. Hyperparameters related to the performance buffer include the number of buffer cells, the maximum number of samples in each buffer cell, the buffer origin, and the tolerance for buffer construction ($\delta_b$). Regarding the Pareto-discovery solver,

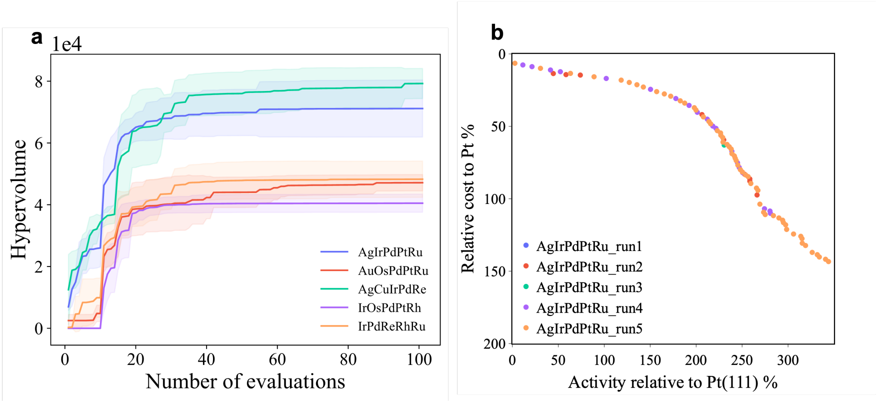


Figure S9: (a) Hypervolume indicator as a function of the number of evaluations, where the curve is averaged over five different seeds, and the variance is represented as a shaded region. For each 5-element space, we conduct multi-objective optimization five times using different random seeds. (b) The learned Pareto fronts for five different runs using bi-objective optimization for catalytic activity and mixing entropy. It is important to note that we consistently discover the same shape of the Pareto front across these runs, and the uncertainty mainly arises from extreme regions where the activity is either too high or too low. Nevertheless, these extreme regions are less interesting for the purpose of discovery.

hyperparameters encompass the factor of perturbation in stochastic sampling ($\delta_p$), the scaling factor for selecting the reference point in local optimization ($\delta_s$), and the number of grid samples on the local manifold. These hyperparameters generally exhibit robustness and have minimal impact on the final performance of multi-objective BO. Increasing the number of buffer cells and the number of grid samples on the local manifold may augment the likelihood of selecting more candidate solutions, albeit with an increased computational cost. The tolerance ($\delta_b$) can influence the error of buffer construction and should be tested. The label cost for the graph cut used in sparsifying the Pareto-front approximation of the batch selection strategy is a hyperparameter that affects clustering. This parameter controls the number of diversity regions (clusters) within the sparse Pareto front.

For the initial samples and batch size per iteration, we used 10 data points and 4 data points for 5-, 6-, and 7-element HEA spaces, and 30 data points and 10 data points for 10-element HEAs space. The larger number of initial samples and batch size used for the 10-element space is due to the fact that there is a much larger design space inhabited.

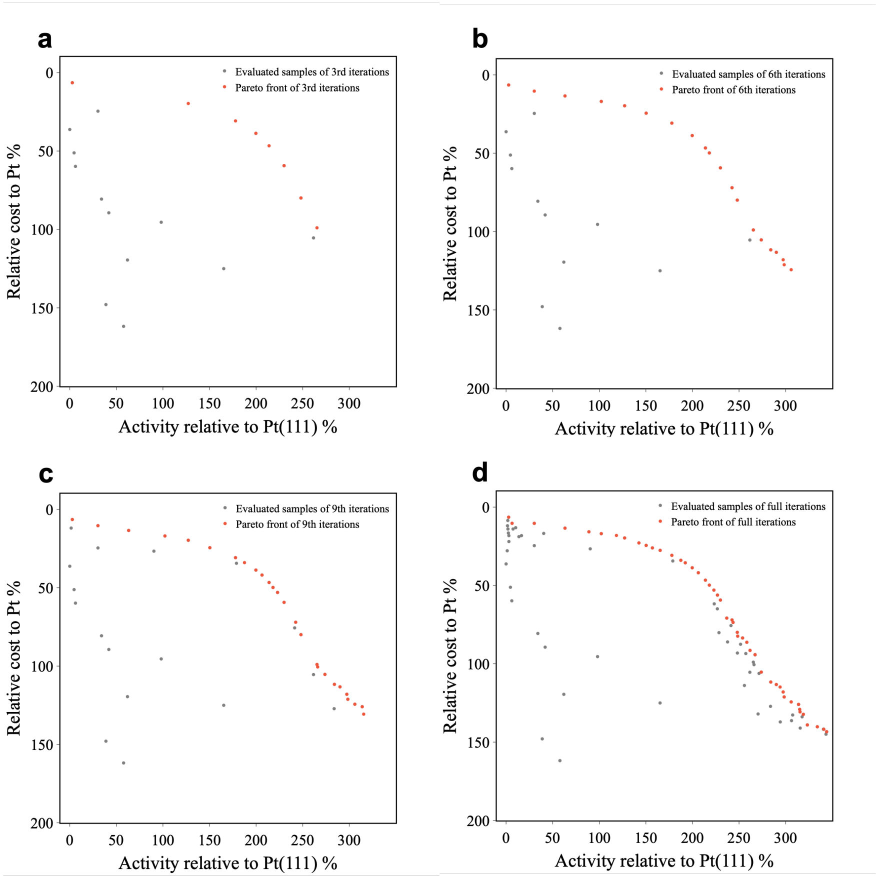


Figure S10: The evolution of learned Pareto front with the increasing number of evaluations up to (a) 3rd iterations, (b) 6th iterations, (c) 9th iterations, and (d) full iterations. For the initial sampling, we used 10 data points, and for each iteration, a batch of 4 data points was suggested. The full iterations refer to the 23rd iteration, which corresponds to a total number of 102 evaluations.

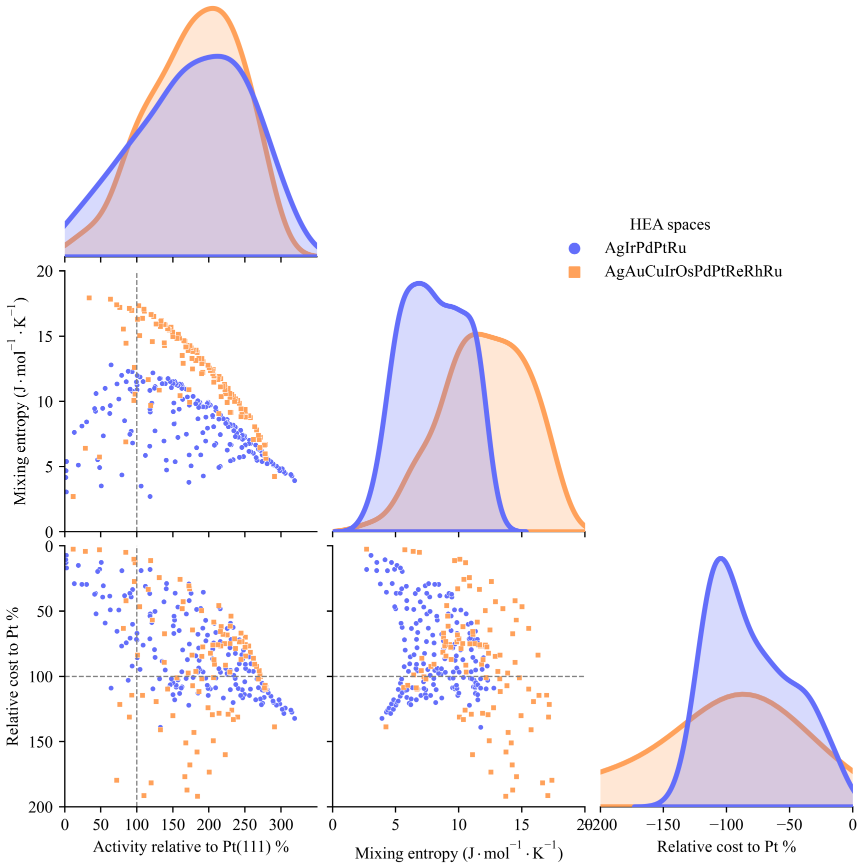


Figure S11: The learned Pareto fronts for a selected 5-element HEA space (AgIrPdPtRu) and a 10-element HEA space (AgAuCuIrOsPdPtReRhRu) using tri-objective optimization for catalytic activity, cost-effectiveness and mixing entropy. The 3D Pareto fronts were projected to three pairwise 2D subplots with estimated probability density for each objective on the off-diagonal. The probability density was normalized independently for each HEA space.

Table S1: Hyperparmaters of the multi-objective BO

| Hyparameter name | Value |
|---|---|
| Number of buffer cells | 100 for 2-dimension, 1000 for higher-dimension |
| Max number of samples in each cell | 10 |
| Buffer origin | $(0,\ldots,0) \in \mathbb{R}^m$ |
| $\delta_b$ | 0.2 |
| $\delta_p$ | 10 |
| $\delta_s$ | 0.3 |
| Label cost for graph cut | 10 |
| Number of grid samples on local manifold | 100 |
| Initial samples | 10 for 5-,6-,7-element HEA spaces, 30 for 10-element HEA space |
| Batch size per iteration | 4 for 5-,6-,7-element HEA spaces, 10 for 10-element HEA space |

## S4.3 Data Efficiency

To quantify the data efficiency of single and multi-objective BO, we conducted single-objective BO using the method described in Ref. 20 and compared them with multi-objective BO results in terms of the number of experiments required to achieve 95% of the optimum performance or 95% of the maximal hypervolume indicator, respectively. The single-objective BO runs target catalytic activity, and these results are presented in Tab. S2, where the number of experiments is averaged over five independent runs for each 5D compositional space. Depending on the compositional space, 15−67 runs are required on average, in reasonable agreement with the number of ca. 50 evaluations reported in Refs. [ 20,21]. For bi-objective optimization in 5D compositional spaces, the number of experiments required is similar to that needed for single-objective BO. Specifically, the results for optimizing catalytic activity and relative cost are comparable (see Tab. S3). Higher-dimensional spaces, such as 6D, 7D, and 10D, require a larger number of evaluations to converge, as indicated in Tab. S4, with approximately 100, 110, and 140 evaluations needed to reach 95% of the maximum hypervolume, respectively. Lastly, tri-objective optimization in the same HEA composition space requires more evaluations than bi-objective optimization, as shown in Tab. S5, with up to 161 evaluations for tri-objective optimization in a 10D compositional space.

Table S2: The number of experiments required to reach 95% of the optimum for single-objective BO on catalytic activity.

| HEA compositional space | Compositional dimensions | No. of experiments |
|---|---|---|
| AgIrPdPtRu | 5D | 30 |
| AuOsPdPtRu | 5D | 67 |
| AgCuIrPdRe | 5D | 30 |
| IrOsPdPtRh | 5D | 48 |
| IrPdReRhRu | 5D | 15 |

Table S3: The number of experiments required to reach 95% of the maximal hypervolume indicator for bi-objective BO on catalytic activity and relative cost.

| HEA compositional space | Compositional dimensions | No. of experiments |
|---|---|---|
| AgIrPdPtRu | 5D | 26 |
| AuOsPdPtRu | 5D | 54 |
| AgCuIrPdRe | 5D | 34 |
| IrOsPdPtRh | 5D | 22 |
| IrPdReRhRu | 5D | 30 |

Table S4: The number of experiments required to reach 95% of the maximal hypervolume indicator for bi-objective BO on catalytic activity and mixing entropy.

| HEA compositional space | Compositional dimensions | No. of experiments |
|---|---|---|
| AgIrPdPtRu | 5D | 53 |
| AuOsPdPtRu | 5D | 58 |
| AgCuIrPdRe | 5D | 30 |
| IrOsPdPtRh | 5D | 70 |
| IrPdReRhRu | 5D | 56 |
| IrOsPdPtRu | 5D | 63 |
| AgIrOsPdPtRu | 6D | 97 |
| AgCuIrOsPdPtRu | 7D | 114 |
| AgAuCuIrOsPdPtReRhRu | 10D | 135 |

Table S5: The number of experiments required to reach 95% of the maximal hypervolume indicator for tri-objective BO on catalytic activity, relative cost and mixing entropy.

| HEA compositional space | Compositional dimensions | No. of experiments |
|---|---|---|
| AgIrPdPtRu | 5D | 94 |
| AgAuCuIrOsPdPtReRhRu | 10D | 161 |

# S5 Additional details on the current density modeling

We use a heuristic current density modeling technique to estimate the average current density per active site (eq. 2) relative to Pt(111) as an ORR activity indicator (as reported in Ref.[1,2,20,31,32]).To obtain net adsorption sites $i$, an explicit simulation that iteratively places adsorbates onto a sufficiently large surface based on adsorption strength was performed while taking into account the co-adsorption behaviors among O* and OH*. The simulation is expedited by a GNN regression model, which enables rapid prediction of adsorption energies in seconds. To ensure adequate surface statistics, we utilize surface supercells of sizes 100 $\times$ 100, 150 $\times$ 150, 200 $\times$ 200, and 250 $\times$ 250 with three atomic layers for the prediction of 5-, 6-, 7-, and 10-element HEA spaces, respectively. The inclusion of three atomic layers is attributed to the consideration of second-rank neighbors in the graph representation of the GNN model. (Further details on net adsorption simulation are provided in section S5.1).

The current density of the net adsorption site $j_i$ is then modeled using the Koutecký-Levich equation (eq. 3), where $j_D$ is the diffusion-limited current (set to -1), and $j_{k,i}$ is the kinetically limiting current obtained from an Arrhenius-like expression (eq. 4). Here, the calculation of the kinetically limiting current requires the consideration of the ORR associative mechanism as described in eqs. 5−8. In eq. 4, $\Delta G_i$ denotes the OH* or O* adsorption free energy, $\Delta G_i^{opt}$ represents the optimal OH* (0.1 eV) or O* (0.2 eV) adsorption free energy larger than these of Pt(111), $c_i$ is a scale factor that is 1 for OH* and 0.5 for O*, U is the applied potential (set to 0.82 V) vs. RHE (reversible hydrogen electrode), $k_B$ is the Boltzmann constant, and $T$ denotes the absolute temperature (set to 298.15 K). The 0.86 eV is the OH* adsorption free energy that ensures simultaneously minimizing the first two steps of ORR: the adsorption of molecular oxygen eq. 5 and the desorption of water eq. 6. Consequently, for the case of the on-top OH* site, assuming the first eq. 5 and last step eq. 8 as the limiting steps, the $j_{k,i}$ is calculated by $-\exp(-\frac{|\Delta G_{\mathrm{OH*}}-\Delta G^{\mathrm{Pt}}_{\mathrm{OH*}}-0.1\mathrm{eV}|-0.86\mathrm{eV}+\mathrm{eU}}{k_BT})$. For the fcc hollow O* site, considering two simultaneous proton and electron transfers to form water (eq. 7 and 8), we can estimate the $j_{k,i}$ in a similar manner as on-top OH* site, that

is, $-\exp(-\frac{0.5\cdot|\Delta G_{O*}-\Delta G^{Pt}_{O*}-0.2\text{eV}|-0.86\text{eV}+\text{eU}}{k_B T})$. For a more detailed explanation and derivation, please refer to sections S5.1, S5.2 and previous publications.[1,2,20]

$$j = \frac{1}{N}\sum_{i=1}^{N} j_i \tag{2}$$

$$\frac{1}{j_i} = \frac{1}{j_D} + \frac{1}{j_{k,i}} \tag{3}$$

$$j_{k,i} = -\exp(-\frac{c_i \cdot |\Delta G_i - \Delta G_i^{opt}| - 0.86\text{eV} + \text{eU}}{k_\text{B} T}) \tag{4}$$

$$1: \quad \text{O}_2\text{(g)} + {}^* + \text{H}^+\text{(aq)} + \text{e}^- \rightleftharpoons \text{OOH}^*, \Delta G_1 = \Delta G_{\text{OOH}*} - 4.92\text{ eV} + \text{eU} \tag{5}$$

$$2: \quad \text{OOH}^* + \text{H}^+\text{(aq)} + \text{e}^- \rightleftharpoons \text{O}^* + \text{H}_2\text{O(l)}, \Delta G_2 = \Delta G_{\text{O}*} - \Delta G_{\text{OOH}*} + \text{eU} \tag{6}$$

$$3: \quad \text{O}^* + \text{H}^+\text{(aq)} + \text{e}^- \rightleftharpoons \text{OH}^*, \Delta G_3 = \Delta G_{\text{OH}*} - \Delta G_{\text{O}*} + \text{eU} \tag{7}$$

$$4: \quad \text{OH}^* + \text{H}^+\text{(aq)} + \text{e}^- \rightleftharpoons \text{H}_2\text{O(l)}, \Delta G_4 = -\Delta G_{\text{OH}*} + \text{eU} \tag{8}$$

## S5.1 Net adsorption enthalpy distribution

In order to get current density, the first step is to obtain a net adsorption enthalpy distribution that is relevant to the actual active site participating in the catalytic reaction. Unlike gross adsorption enthalpy distribution, which simply includes all sites available on the surface, net adsorption enthalpy distribution accounts for active sites affected by co-adsorption interplay between O* and OH* at certain applied potential. It is obtained by performing an explicit simulation that iteratively places adsorbates onto the surface based on their adsorption strength. To model the effect of an applied electrode potential U vs. the reversible hydrogen electrode (RHE), we express the Gibbs free energy of OH* and O* as $\Delta G_{\text{OH}*}(\text{U}) = \Delta G_{\text{OH}*}(0) - \text{eU}$ and $\Delta G_{\text{O}*}(\text{U}) = \Delta G_{\text{O}*}(0) - 2\text{eU}$.[33] This is because O* has to undergo

two proton-coupled electron transfer (PCET) steps but OH* only one. If we let $\Delta G_{\mathrm{OH}*}$ and $\Delta G_{\mathrm{O}*}$ equal to 0, it means that adsorption starts happening at this time, thereby we have $\Delta G_{\mathrm{OH}*}(0) = \frac{1}{2}\Delta G_{\mathrm{O}*}(0) = \mathrm{eU}$. In this way, when U is increased, we can compute $\Delta G_{\mathrm{OH}*}(0)$ and $\frac{1}{2}\Delta G_{\mathrm{O}*}(0)$ with respect to eU to determine the occurrence of adsorption, that is, whether active sites are occupied or not.

At the starting point of the explicit simulation, a bare HEA surface is represented by a sufficiently large fcc(111) supercell, e.g., $100 \times 100$, for the sake of adequate surface statistics. Based on the way of determining adsorption described above, we can look at all active sites available on the bare surface with the help of the ML regression model and then place the first adsorbate on the strongest binding site. At the next interaction, some restriction rules are imposed to mimic competitive co-adsorption between O* and OH*, ensuring that no surface atom is bonded to more than one adsorbate. More precisely, these rules include: 1) An adsorbed on-top OH* will obstruct the adjacent three fcc hollow sites. 2) If two adsorbed on-top OH* molecules form an immediate neighbor, the adjacent five fcc hollow sites and the two shared on-top sites will be obstructed. 3) An adsorbed fcc O* will obstruct the adjacent three on-top sites and six neighboring fcc hollow sites (see Fig. S12). Under these rules, the second strongest binding sites will be occupied accordingly. This procedure is executed iteratively until no sites are available.

## S5.2 Modeling reactivity of oxygen reduction reaction (ORR)

The ORR is a very sluggish reaction represented by eq. 9 with a reduction potential of +1.23 V vs. the reversible hydrogen electrode (RHE).[34]

$$\mathrm{O_2(g) + 4\,(H^+(aq) + e^-) \rightleftharpoons 2\,H_2O(l)} \tag{9}$$

We consider and express the associative mechanism as the sequence of the four elementary PCET steps as described in eqs. 5–8, where $\Delta G_i$ is the Gibbs free energy change (reaction

energy) for each step, e is the elementary charge, U is the electrochemical potential (vs. RHE). In order to get a reaction rate, we use an Arrhenius expression $r = k \cdot \exp \frac{-\Delta G^{\ddagger}_{\mathrm{RLS}}}{k_{\mathrm{B}}T}$, in which k is a pre-exponential factor, $\Delta G^{\ddagger}_{\mathrm{RLS}}$ is the Gibbs free energy changes of the transition state of the rate-limiting step, $k_{\mathrm{B}}$ is the Boltzmann constant, and T is the temperature (set to 298.15 K).

Since the electron-proton transfers in these reactions are generally facile,[35] the reaction barriers are often neglected so that the relevant $\Delta G^{\ddagger}_{\mathrm{RLS}}$ is equal to the reaction energy of the most uphill elementary reaction: $\Delta G^{\ddagger}_{\mathrm{RLS}} \approx \max(\Delta G_1, \Delta G_2, \Delta G_3, \Delta G_4)$. We therefore need to express the Gibbs free energy changes of the rate-limiting step in terms of adsorption enthalpies of the key intermediates. It has been proved that in HEAs, there are same-site binding energy linear scaling relations between on-top OOH* and on-top OH*,[36,37] such that $\Delta G_{\mathrm{OOH}*}$ can be approximated as $\Delta G_{\mathrm{OOH}*} = \Delta G_{\mathrm{OH}*} + 3.2$ eV. We then rewrite the first step of the associative mechanism to be $\Delta G_1 = \Delta G_{\mathrm{OH}*} - 1.72$ eV + eU. Considering only the first and the final step to be rate limiting[33,38] yields an expression only depending on the adsorption enthalpy of OH*, that is, $\max(\Delta G_1, \Delta G_4) = \max(\Delta G_{\mathrm{OH}*} - 1.72\ \mathrm{eV} + \mathrm{eU}, -\Delta G_{\mathrm{OH}*} + \mathrm{eU})$ $= |\Delta G_{\mathrm{OH}*} - 0.86\ \mathrm{eV}| - 0.86\ \mathrm{eV} + \mathrm{eU}$. Therefore, using Arrhenius expression and replacing $\Delta G^{\ddagger}_{\mathrm{RLS}}$, we can now express the reaction rate as $r_{\mathrm{OH}*} = k \cdot \exp(-\frac{|\Delta G_{\mathrm{OH}*} - 0.86\ \mathrm{eV}| - 0.86\ \mathrm{eV} + \mathrm{eU}}{k_{\mathrm{B}}T})$. Note that the optimum catalytic activity at an adsorption enthalpy of 0.86 eV has been observed to be 0.1 and 0.2 eV larger than OH* and O* on Pt(111).[39,40] We can further rewrite the reaction rate to $r_{\mathrm{OH}*} = k \cdot \exp(-\frac{|\Delta G_{\mathrm{OH}*} - \Delta G^{\mathrm{Pt}}_{\mathrm{OH}*} - 0.1\ \mathrm{eV}| - 0.86\ \mathrm{eV} + \mathrm{eU}}{k_{\mathrm{B}}T})$ relative to a pure Pt(111) surface. By doing so, there is no need to include the additional correction of the electrochemical environment (such as solvation stabilization) to the Gibbs free energy changes, as it is assumed to have the same effect as Pt(111) and therefore cancels out.

To determine the reaction rate of fcc hollow O* sites, we consider the last two PCET steps to the formation of water to serve as an upper bound for the activity of fcc hollow O* sites. We assume that the O* adsorption enthalpy will scale with the OH* adsorption enthalpy for active sites, with a slope of 2, i.e. $\Delta G_{\mathrm{O}*} = 2\Delta G_{\mathrm{OH}*}$ (also see section S5.1).

To compute the activity of fcc hollow O* sites using an expression similar to OH*, we get $r_{\mathrm{O}*} = k \cdot \exp(-\frac{0.5|\Delta G_{\mathrm{O}*} - \Delta G_{\mathrm{O}*}^{\mathrm{Pt}} - 0.2\ \mathrm{eV}| - 0.86\ \mathrm{eV} + \mathrm{eU}}{k_{\mathrm{B}}T})$.

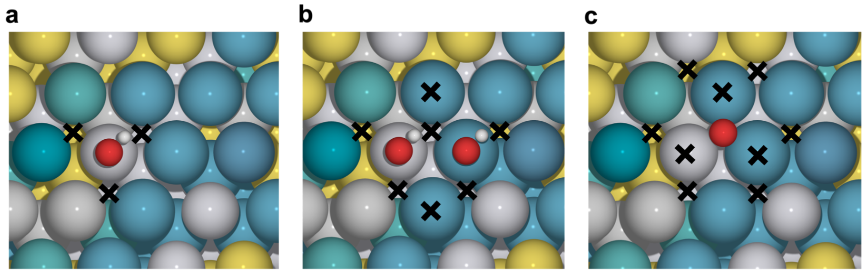


Figure S12: Illustration of three blocking rules during explicit simulation of the heuristic current density modeling. (a) An adsorbed on-top OH* will obstruct the adjacent three fcc hollow sites. (b) If two adsorbed on-top OH* molecules form an immediate neighbor, the adjacent five fcc hollow sites and the two shared on-top sites will be obstructed. (c) An adsorbed fcc O* will obstruct three adjacent on-top sites and six neighboring fcc hollow sites. Redrawn from Fig. 6 in Ref. 1.

# S6 Correlated mixing entropy

While ideal mixing entropy is widespread use in HEA studies, it has been proposed that the formation of a random solid solution is influenced not solely by $S_{id}$ but also by other factors like atomic size difference and mixing enthalpy of the constituent atomic pairs.[41,42] Additionally, the occurrence of metastability in the random solid solution has been noted at lower temperatures. In our pursuit to move beyond ideal mixing entropy, we performed an empirical technique known as correlated mixing entropy, introduced by Yang et al.,[43,44] which takes into consideration both atomic size and chemical bond misfit.

Its general structure can be formulated as $S_{corr} = S_{id} + S_E$. In this context, $S_{id}$ signifies the ideal mixing entropy, while $S_E$ represents the excessive entropy of mixing due to correlation. Drawing from statistical thermodynamics and considering the general effect of potential energy fluctuations, Yang et al. derived a $S_E$ formula, eq. 10, in which $x$ is the normalized energy fluctuation, encompassing contributions from two sources, namely atom size misfit ($x_e$) and chemical bond misfit ($x_c$) as outlined in eq. 11. Furthermore, according

to references,[43,44] $x_e$ and $x_c$ can be formulated as eq. 12 and eq. 13 respectively, where $\bar{K}$ is the average bulk modulus, $\bar{V}$ is the average atomic volume, $H_{ij}$ denotes the mixing enthalpy between the $i_{th}$ and $j_{th}$ elements, and $\bar{H}$ indicates the average of $H_{ij}$. These tabulated features were obtained from.[45,46] For a detailed explanation of the correlated mixing entropy derivation, readers are directed to the original publications.[41,43,44]

Leveraging this correlated mixing entropy, we conducted bi-objective optimizations for catalytic activity and correlated mixing entropy across five 5- to 10-element HEA spaces at temperatures of 1000 K and 2000 K. The resulting learned Pareto fronts are displayed in Fig. S13. A similar trend is evident when compared with the learned Pareto front of catalytic activity and ideal mixing entropy (see Fig. 4 in the main text). Notably, there are more pronounced gaps among these HEA spaces at 2000 K, suggesting that the entropic effect becomes more significant with an increasing number of elements within HEAs.

$$S_E = k_{\mathrm{B}} \times \left[1 + \frac{x}{2} - \ln(x) + \ln(1 - e^{-x}) - \frac{x}{2} \times \frac{1 + e^{-x}}{1 - e^{-x}}\right] \tag{10}$$

$$x = x_e + x_c \tag{11}$$

$$x_e = 4.12\delta \times \sqrt{\frac{\bar{K}\bar{V}}{k_{\mathrm{B}}T}} \tag{12}$$

$$x_c = 2\sqrt{\frac{\sqrt{\sum_i \sum_{j,i\neq j} c_i c_j (H_{ij} - \bar{H})^2}}{k_{\mathrm{B}}T}} \tag{13}$$

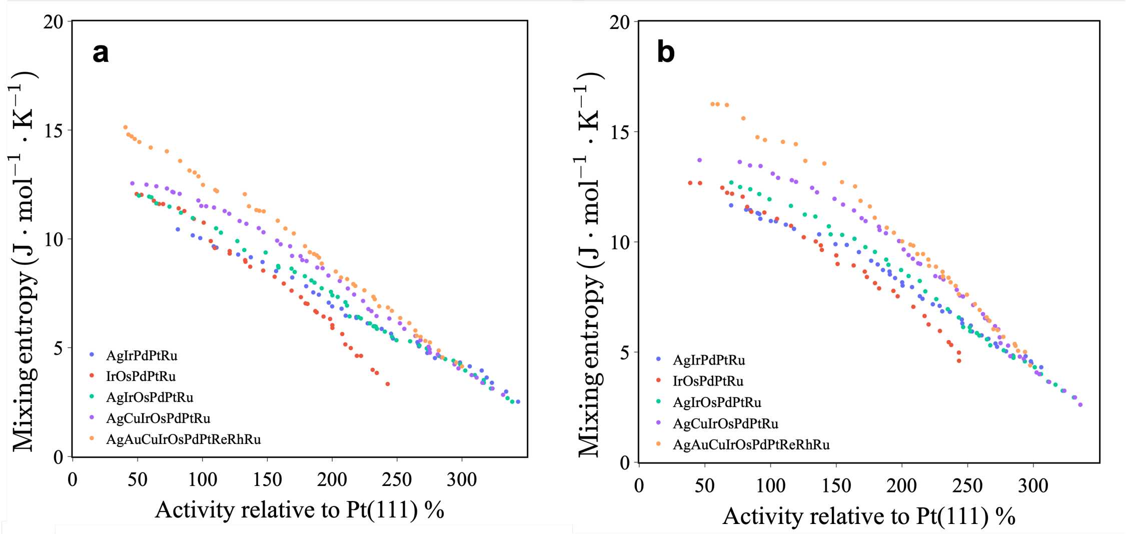


Figure S13: The learned Pareto fronts for five 5- to 10-element HEA spaces using bi-objective optimization for catalytic activity and correlated mixing entropy at temperatures of (a) 1000 K and (b) 2000 K

# S7 Full list of Pareto-optimal solutions

Table S6: Pareto-optimal solutions found by tri-objective optimization for 10-element HEA space (AgAuCuIrOsPdPtReRhRu) within 300 evaluations. Displayed are three objectives: catalytic activity (in %), mixing entropy (in $J \cdot mol^{-1} \cdot K^{-1}$), and relative cost (in %), alongside the individual fraction of constituent elements (in %).

| Catalytic activity | Mixing entropy | Relative cost | Ag | Au | Cu | Ir | Os | Pd | Pt | Re | Rh | Ru |
|---|---|---|---|---|---|---|---|---|---|---|---|---|
| 291.08 | 4.24 | 138.70 | 11 | 0 | 0 | 0 | 0 | 85 | 0 | 0 | 0 | 4 |
| 279.29 | 5.65 | 108.46 | 32 | 0 | 1 | 0 | 0 | 67 | 0 | 0 | 0 | 0 |
| 278.58 | 6.71 | 109.89 | 24 | 0 | 8 | 0 | 0 | 68 | 0 | 0 | 0 | 0 |
| 278.24 | 5.96 | 106.85 | 32 | 0 | 2 | 0 | 0 | 66 | 0 | 0 | 0 | 0 |
| 277.17 | 6.65 | 108.81 | 28 | 0 | 4 | 0 | 0 | 67 | 0 | 0 | 0 | 1 |
| 271.58 | 7.25 | 100.35 | 29 | 0 | 9 | 0 | 0 | 62 | 0 | 0 | 0 | 0 |
| 270.94 | 6.44 | 98.90 | 36 | 0 | 3 | 0 | 0 | 61 | 0 | 0 | 0 | 0 |
| 269.96 | 7.05 | 95.66 | 35 | 0 | 6 | 0 | 0 | 59 | 0 | 0 | 0 | 0 |
| 269.01 | 7.40 | 102.08 | 30 | 1 | 7 | 0 | 0 | 62 | 0 | 0 | 0 | 0 |
| 266.35 | 7.80 | 92.36 | 31 | 0 | 12 | 0 | 0 | 57 | 0 | 0 | 0 | 0 |
| 263.66 | 8.05 | 90.70 | 29 | 0 | 15 | 0 | 0 | 56 | 0 | 0 | 0 | 0 |
| 261.92 | 7.07 | 86.17 | 42 | 0 | 5 | 0 | 0 | 53 | 0 | 0 | 0 | 0 |
| 257.64 | 8.74 | 81.59 | 33 | 0 | 16 | 0 | 0 | 50 | 0 | 0 | 0 | 1 |
| 255.15 | 8.89 | 76.81 | 35 | 0 | 17 | 0 | 0 | 47 | 0 | 0 | 0 | 1 |
| 250.84 | 8.99 | 84.54 | 34 | 0 | 11 | 0 | 0 | 51 | 0 | 0 | 0 | 4 |
| 250.75 | 8.80 | 69.96 | 36 | 0 | 21 | 0 | 0 | 43 | 0 | 0 | 0 | 0 |
| 250.54 | 9.29 | 75.60 | 33 | 0 | 19 | 0 | 0 | 46 | 0 | 0 | 0 | 2 |
| 250.22 | 9.37 | 77.65 | 33 | 0 | 17 | 0 | 0 | 47 | 0 | 0 | 0 | 3 |
| 245.95 | 8.98 | 73.32 | 39 | 0 | 14 | 0 | 0 | 45 | 0 | 2 | 0 | 0 |
| 244.72 | 8.81 | 66.94 | 43 | 0 | 15 | 0 | 0 | 41 | 0 | 1 | 0 | 0 |
| 244.70 | 9.41 | 87.09 | 31 | 0 | 12 | 0 | 0 | 52 | 1 | 0 | 0 | 4 |

| | | | | | | | | | | | | |
|---|---|---|---|---|---|---|---|---|---|---|---|---|
| 242.23 | 9.86 | 73.71 | 33 | 0 | 18 | 0 | 0 | 44 | 0 | 0 | 0 | 5 |
| 239.98 | 10.06 | 131.26 | 32 | 1 | 16 | 0 | 1 | 47 | 0 | 0 | 0 | 3 |
| 236.87 | 10.19 | 78.70 | 31 | 1 | 18 | 0 | 0 | 46 | 1 | 0 | 0 | 3 |
| 235.85 | 10.01 | 71.21 | 35 | 0 | 19 | 0 | 0 | 42 | 2 | 0 | 0 | 2 |
| 234.01 | 10.37 | 133.15 | 25 | 1 | 21 | 0 | 1 | 48 | 0 | 0 | 0 | 4 |
| 231.89 | 10.51 | 125.92 | 26 | 0 | 23 | 0 | 1 | 44 | 0 | 0 | 0 | 6 |
| 230.59 | 9.13 | 53.84 | 34 | 0 | 33 | 0 | 0 | 33 | 0 | 0 | 0 | 0 |
| 229.81 | 9.16 | 61.59 | 15 | 1 | 46 | 0 | 0 | 37 | 0 | 1 | 0 | 0 |
| 229.51 | 10.32 | 74.85 | 25 | 2 | 26 | 0 | 0 | 44 | 0 | 3 | 0 | 0 |
| 229.14 | 10.60 | 96.75 | 21 | 1 | 21 | 0 | 0 | 51 | 0 | 3 | 1 | 2 |
| 226.77 | 11.00 | 129.02 | 29 | 1 | 19 | 0 | 1 | 45 | 1 | 1 | 0 | 3 |
| 225.51 | 9.91 | 68.41 | 21 | 4 | 37 | 0 | 0 | 38 | 0 | 0 | 0 | 0 |
| 222.93 | 10.57 | 91.38 | 18 | 0 | 15 | 0 | 0 | 54 | 0 | 4 | 0 | 9 |
| 222.65 | 11.09 | 160.14 | 28 | 0 | 19 | 0 | 1 | 45 | 0 | 3 | 3 | 1 |
| 222.52 | 10.88 | 75.17 | 24 | 1 | 26 | 0 | 0 | 43 | 2 | 0 | 0 | 4 |
| 221.61 | 11.29 | 74.95 | 22 | 2 | 26 | 0 | 0 | 43 | 0 | 3 | 0 | 4 |
| 219.78 | 11.14 | 67.80 | 27 | 3 | 28 | 0 | 0 | 38 | 0 | 2 | 0 | 2 |
| 217.24 | 10.35 | 61.85 | 15 | 2 | 42 | 0 | 0 | 36 | 0 | 5 | 0 | 0 |
| 216.39 | 11.72 | 76.79 | 22 | 2 | 24 | 0 | 0 | 43 | 1 | 2 | 0 | 6 |
| 216.27 | 11.11 | 65.60 | 32 | 4 | 25 | 0 | 0 | 35 | 0 | 0 | 0 | 4 |
| 214.07 | 11.89 | 128.39 | 27 | 2 | 19 | 0 | 1 | 43 | 1 | 2 | 0 | 5 |
| 213.89 | 9.06 | 45.77 | 33 | 0 | 39 | 0 | 0 | 28 | 0 | 0 | 0 | 0 |
| 212.39 | 11.67 | 75.81 | 20 | 2 | 29 | 1 | 0 | 40 | 0 | 1 | 0 | 7 |
| 207.98 | 11.81 | 90.71 | 30 | 2 | 23 | 3 | 0 | 38 | 0 | 2 | 1 | 1 |
| 207.14 | 11.94 | 87.20 | 25 | 3 | 26 | 1 | 0 | 39 | 1 | 0 | 1 | 4 |
| 206.85 | 11.47 | 58.45 | 32 | 4 | 28 | 0 | 0 | 30 | 0 | 0 | 0 | 6 |
| 206.56 | 12.17 | 100.94 | 20 | 2 | 22 | 0 | 0 | 44 | 0 | 3 | 2 | 7 |

| | | | | | | | | | | | | |
|---|---|---|---|---|---|---|---|---|---|---|---|---|
| 206.29 | 12.42 | 149.40 | 25 | 2 | 21 | 0 | 1 | 42 | 1 | 2 | 2 | 4 |
| 205.57 | 12.13 | 76.04 | 27 | 3 | 25 | 2 | 0 | 37 | 0 | 2 | 0 | 4 |
| 204.99 | 12.34 | 129.46 | 18 | 3 | 24 | 0 | 1 | 43 | 0 | 5 | 0 | 6 |
| 202.03 | 12.37 | 82.26 | 21 | 2 | 25 | 0 | 0 | 39 | 0 | 3 | 1 | 9 |
| 200.44 | 12.56 | 173.48 | 25 | 4 | 24 | 0 | 2 | 37 | 0 | 2 | 0 | 6 |
| 198.91 | 12.77 | 124.05 | 21 | 3 | 25 | 0 | 1 | 39 | 1 | 4 | 0 | 6 |
| 192.00 | 13.04 | 326.52 | 25 | 2 | 16 | 0 | 4 | 42 | 0 | 5 | 4 | 2 |
| 191.62 | 12.23 | 77.54 | 16 | 0 | 23 | 0 | 0 | 43 | 3 | 4 | 0 | 11 |
| 190.38 | 12.89 | 84.75 | 24 | 5 | 23 | 3 | 0 | 37 | 0 | 2 | 0 | 6 |
| 190.18 | 13.26 | 101.88 | 23 | 3 | 21 | 1 | 0 | 40 | 2 | 3 | 2 | 5 |
| 189.94 | 12.11 | 49.69 | 33 | 3 | 29 | 0 | 0 | 25 | 0 | 2 | 0 | 8 |
| 186.25 | 13.29 | 111.63 | 28 | 2 | 16 | 1 | 0 | 39 | 3 | 2 | 3 | 6 |
| 184.28 | 13.73 | 192.05 | 20 | 4 | 24 | 1 | 2 | 38 | 1 | 3 | 1 | 6 |
| 183.71 | 13.75 | 158.11 | 23 | 3 | 22 | 3 | 1 | 38 | 1 | 3 | 2 | 4 |
| 182.13 | 13.93 | 215.88 | 21 | 3 | 20 | 1 | 2 | 40 | 1 | 5 | 3 | 4 |
| 181.92 | 13.11 | 96.27 | 27 | 1 | 29 | 2 | 0 | 30 | 1 | 5 | 3 | 2 |
| 179.31 | 13.93 | 143.03 | 20 | 4 | 24 | 2 | 1 | 37 | 1 | 4 | 1 | 6 |
| 177.46 | 13.83 | 107.19 | 25 | 4 | 22 | 4 | 0 | 35 | 1 | 4 | 2 | 3 |
| 177.34 | 12.49 | 64.45 | 34 | 0 | 15 | 0 | 0 | 34 | 7 | 6 | 0 | 4 |
| 176.65 | 13.28 | 83.68 | 15 | 5 | 20 | 0 | 0 | 41 | 3 | 3 | 0 | 13 |
| 175.23 | 14.10 | 265.13 | 19 | 5 | 21 | 3 | 3 | 39 | 0 | 6 | 2 | 2 |
| 173.81 | 14.25 | 168.01 | 21 | 5 | 19 | 4 | 1 | 39 | 1 | 3 | 2 | 5 |
| 173.40 | 9.92 | 33.00 | 26 | 0 | 48 | 0 | 0 | 20 | 0 | 6 | 0 | 0 |
| 172.39 | 10.88 | 30.74 | 42 | 0 | 29 | 0 | 0 | 18 | 0 | 10 | 0 | 1 |
| 171.42 | 13.89 | 97.45 | 17 | 3 | 24 | 1 | 0 | 36 | 1 | 4 | 2 | 12 |
| 169.57 | 14.28 | 187.24 | 21 | 4 | 19 | 4 | 1 | 38 | 0 | 3 | 4 | 6 |
| 168.15 | 14.16 | 151.00 | 27 | 3 | 26 | 2 | 1 | 29 | 2 | 5 | 3 | 2 |

| | | | | | | | | | | | | |
|---|---|---|---|---|---|---|---|---|---|---|---|---|
| 166.57 | 14.59 | 176.85 | 21 | 4 | 19 | 4 | 1 | 38 | 2 | 4 | 3 | 4 |
| 165.36 | 14.65 | 277.29 | 19 | 6 | 15 | 2 | 3 | 40 | 0 | 6 | 3 | 6 |
| 164.24 | 12.92 | 42.93 | 33 | 7 | 31 | 0 | 0 | 17 | 0 | 6 | 0 | 6 |
| 162.90 | 13.50 | 48.09 | 31 | 3 | 28 | 0 | 0 | 22 | 4 | 6 | 0 | 6 |
| 160.07 | 10.42 | 25.54 | 43 | 0 | 31 | 0 | 0 | 15 | 0 | 11 | 0 | 0 |
| 159.32 | 14.85 | 222.50 | 23 | 4 | 21 | 4 | 2 | 34 | 3 | 4 | 3 | 2 |
| 157.74 | 15.14 | 212.65 | 18 | 5 | 21 | 3 | 2 | 36 | 3 | 5 | 2 | 5 |
| 155.60 | 14.82 | 101.18 | 20 | 4 | 19 | 4 | 0 | 35 | 4 | 4 | 1 | 9 |
| 150.09 | 14.95 | 130.19 | 17 | 9 | 20 | 4 | 0 | 36 | 2 | 5 | 3 | 4 |
| 149.75 | 15.31 | 213.78 | 18 | 6 | 18 | 0 | 2 | 35 | 5 | 6 | 3 | 7 |
| 147.28 | 15.68 | 329.55 | 17 | 6 | 20 | 1 | 4 | 34 | 3 | 5 | 4 | 6 |
| 144.96 | 15.47 | 280.25 | 18 | 3 | 18 | 0 | 3 | 33 | 3 | 6 | 5 | 11 |
| 142.80 | 15.56 | 109.75 | 20 | 6 | 19 | 4 | 0 | 31 | 4 | 5 | 2 | 9 |
| 139.27 | 15.85 | 278.80 | 13 | 3 | 21 | 4 | 3 | 35 | 5 | 5 | 3 | 8 |
| 138.45 | 14.28 | 67.40 | 20 | 4 | 17 | 0 | 0 | 29 | 4 | 5 | 0 | 21 |
| 138.21 | 14.48 | 89.18 | 24 | 9 | 25 | 7 | 0 | 24 | 3 | 8 | 0 | 0 |
| 137.97 | 15.88 | 324.37 | 20 | 8 | 17 | 0 | 4 | 31 | 4 | 7 | 4 | 5 |
| 134.23 | 16.18 | 221.38 | 18 | 7 | 18 | 3 | 2 | 31 | 3 | 6 | 3 | 9 |
| 133.51 | 16.14 | 218.40 | 16 | 6 | 20 | 5 | 2 | 31 | 4 | 4 | 2 | 10 |
| 132.93 | 15.75 | 140.98 | 17 | 5 | 16 | 3 | 0 | 32 | 3 | 5 | 5 | 14 |
| 131.17 | 10.86 | 24.35 | 42 | 0 | 22 | 0 | 0 | 14 | 0 | 22 | 0 | 0 |
| 126.71 | 15.93 | 119.28 | 15 | 6 | 20 | 6 | 0 | 30 | 5 | 4 | 2 | 12 |
| 123.54 | 16.50 | 293.03 | 17 | 6 | 19 | 3 | 3 | 28 | 5 | 2 | 5 | 12 |
| 121.72 | 14.57 | 56.88 | 21 | 11 | 22 | 0 | 0 | 20 | 0 | 15 | 0 | 11 |
| 120.03 | 16.65 | 181.71 | 17 | 8 | 17 | 3 | 1 | 28 | 6 | 5 | 4 | 11 |
| 119.11 | 9.68 | 11.36 | 49 | 0 | 30 | 0 | 0 | 6 | 0 | 15 | 0 | 0 |
| 116.05 | 11.65 | 33.31 | 15 | 0 | 40 | 0 | 0 | 19 | 1 | 24 | 0 | 1 |

| | | | | | | | | | | | | |
|---|---|---|---|---|---|---|---|---|---|---|---|---|
| 110.51 | 16.92 | 218.65 | 18 | 6 | 17 | 8 | 1 | 27 | 5 | 7 | 6 | 5 |
| 109.95 | 17.07 | 191.37 | 15 | 7 | 20 | 4 | 1 | 25 | 7 | 7 | 5 | 9 |
| 107.98 | 16.38 | 114.51 | 18 | 13 | 18 | 3 | 0 | 23 | 2 | 10 | 3 | 10 |
| 104.06 | 15.02 | 46.78 | 27 | 3 | 19 | 0 | 0 | 15 | 9 | 10 | 0 | 17 |
| 102.55 | 17.32 | 202.72 | 14 | 9 | 18 | 6 | 1 | 25 | 6 | 7 | 5 | 9 |
| 100.90 | 16.30 | 83.71 | 18 | 9 | 15 | 4 | 0 | 23 | 6 | 10 | 0 | 15 |
| 97.61 | 10.55 | 13.03 | 32 | 0 | 35 | 0 | 0 | 7 | 0 | 26 | 0 | 0 |
| 96.68 | 12.59 | 27.22 | 29 | 0 | 24 | 0 | 0 | 9 | 11 | 27 | 0 | 0 |
| 96.17 | 10.09 | 10.26 | 41 | 0 | 22 | 0 | 0 | 5 | 0 | 32 | 0 | 0 |
| 92.96 | 16.98 | 114.48 | 16 | 8 | 18 | 9 | 0 | 20 | 13 | 8 | 1 | 7 |
| 89.76 | 17.12 | 131.17 | 18 | 10 | 16 | 10 | 0 | 20 | 9 | 5 | 2 | 10 |
| 84.94 | 14.47 | 42.16 | 15 | 0 | 24 | 0 | 0 | 13 | 16 | 23 | 0 | 9 |
| 84.62 | 6.89 | 4.89 | 67 | 0 | 27 | 0 | 0 | 2 | 0 | 4 | 0 | 0 |
| 81.45 | 15.55 | 63.28 | 12 | 6 | 20 | 0 | 0 | 14 | 15 | 26 | 1 | 6 |
| 76.55 | 17.19 | 121.59 | 19 | 13 | 13 | 8 | 0 | 16 | 12 | 13 | 2 | 4 |
| 72.10 | 17.40 | 179.72 | 18 | 14 | 10 | 11 | 1 | 19 | 9 | 11 | 1 | 6 |
| 63.46 | 17.84 | 340.96 | 19 | 10 | 5 | 6 | 2 | 20 | 6 | 10 | 13 | 9 |
| 48.23 | 5.74 | 3.07 | 54 | 0 | 0 | 0 | 0 | 0 | 0 | 46 | 0 | 0 |
| 33.92 | 17.92 | 927.31 | 18 | 7 | 9 | 8 | 15 | 6 | 8 | 20 | 7 | 2 |
| 28.74 | 6.40 | 4.19 | 43 | 0 | 55 | 0 | 0 | 2 | 0 | 0 | 0 | 0 |
| 11.87 | 2.70 | 2.42 | 90 | 0 | 0 | 0 | 0 | 0 | 0 | 10 | 0 | 0 |

# References

(1) Clausen, C. M.; Nielsen, M. L. S.; Pedersen, J. K.; Rossmeisl, J. Ab Initio to Activity: Machine Learning-Assisted Optimization of High-Entropy Alloy Catalytic Activity. *High Entropy Alloys & Materials* **2023**, *1*, 120–133.

(2) Batchelor, T. A. A.; Löffler, T.; Xiao, B.; Krysiak, O. A.; Strotkötter, V.; Pedersen, J. K.; Clausen, C. M.; Savan, A.; Li, Y.; Schuhmann, W.; Rossmeisl, J.; Ludwig, A. Complex-Solid-Solution Electrocatalyst Discovery by Computational Prediction and High-Throughput Experimentation**. *Angew. Chem. Int. Ed.* **2021**, *60*, 6932–6937.

(3) Mints, V.; Pedersen, J. K.; Wiberg, G. K. H.; Rossmeisl, J.; Arenz, M. *Backward Elimination: A Strategy for High-Entropy Alloy Catalyst Discovery*; Preprint at 10.26434/chemrxiv-2022-78s83, 2022.

(4) Du, C.; Gao, X.; Chen, W. Recent Developments in Copper-Based, Non-Noble Metal Electrocatalysts for the Oxygen Reduction Reaction. *Chinese J. Catal.* **2016**, *37*, 1049–1061.

(5) Kim, C.; Dionigi, F.; Beermann, V.; Wang, X.; Möller, T.; Strasser, P. Alloy Nanocatalysts for the Electrochemical Oxygen Reduction (ORR) and the Direct Electrochemical Carbon Dioxide Reduction Reaction ($CO_2$RR). *Adv. Mater.* **2019**, *31*, 1805617.

(6) Enkovaara, J. et al. Electronic Structure Calculations with GPAW: A Real-Space Implementation of the Projector Augmented-Wave Method. *J. Phys.: Condens. Matter* **2010**, *22*, 253202.

(7) Mortensen, J. J.; Hansen, L. B.; Jacobsen, K. W. Real-Space Grid Implementation of the Projector Augmented Wave Method. *Phys. Rev. B* **2005**, *71*, 035109.

(8) Hammer, B.; Hansen, L. B.; Nørskov, J. K. Improved Adsorption Energetics within

Density-Functional Theory Using Revised Perdew-Burke-Ernzerhof Functionals. *Phys. Rev. B* **1999**, *59*, 7413–7421.

(9) Clausen, C. M.; Pedersen, J. K.; Batchelor, T. A. A.; Rossmeisl, J. Lattice Distortion Releasing Local Surface Strain on High-Entropy Alloys. *Nano Res.* **2022**, *15*, 4775–4779.

(10) Clausen, C. M.; Batchelor, T. A. A.; Pedersen, J. K.; Rossmeisl, J. What Atomic Positions Determines Reactivity of a Surface? Long-Range, Directional Ligand Effects in Metallic Alloys. *Adv. Sci.* **2021**, *8*, 2003357.

(11) Hjorth Larsen, A. et al. The Atomic Simulation Environment—a Python Library for Working with Atoms. *J. Phys.: Condens. Matter* **2017**, *29*, 273002.

(12) Gasteiger, J.; Shuaibi, M.; Sriram, A.; Günnemann, S.; Ulissi, Z.; Zitnick, C. L.; Das, A. GemNet-OC: Developing Graph Neural Networks for Large and Diverse Molecular Simulation Datasets. 2022.

(13) Zhang, J.; Wang, C.; Huang, S.; Xiang, X.; Xiong, Y.; Xu, B.; Ma, S.; Fu, H.; Kai, J.; Kang, X.; Zhao, S. Design High-Entropy Electrocatalyst via Interpretable Deep Graph Attention Learning. *Joule* **2023**, *7*, 1832–1851.

(14) Andersen, M.; Levchenko, S. V.; Scheffler, M.; Reuter, K. Beyond Scaling Relations for the Description of Catalytic Materials. *ACS Catal.* **2019**, *9*, 2752–2759.

(15) Zong, X.; Vlachos, D. G. Exploring Structure-Sensitive Relations for Small Species Adsorption Using Machine Learning. *J. Chem. Inf. Model.* **2022**, *62*, 4361–4368.

(16) Li, Y.; Tarlow, D.; Brockschmidt, M.; Zemel, R. Gated graph sequence neural networks. *arXiv preprint arXiv:1511.05493* **2015**,

(17) Chanussot, L. et al. Open Catalyst 2020 (OC20) Dataset and Community Challenges. *ACS Catal.* **2021**, *11*, 6059–6072.

(18) Konakovic Lukovic, M.; Tian, Y.; Matusik, W. Diversity-guided multi-objective bayesian optimization with batch evaluations. *Adv. Neural Inf. Process. Syst.* **2020**, *33*, 17708–17720.

(19) Schulz, A.; Wang, H.; Grinspun, E.; Solomon, J.; Matusik, W. Interactive Exploration of Design Trade-Offs. *ACM Trans. Graph.* **2018**, *37*, 1–14.

(20) Pedersen, J. K.; Clausen, C. M.; Krysiak, O. A.; Xiao, B.; Batchelor, T. A. A.; Löffler, T.; Mints, V. A.; Banko, L.; Arenz, M.; Savan, A.; Schuhmann, W.; Ludwig, A.; Rossmeisl, J. Bayesian Optimization of High-Entropy Alloy Compositions for Electrocatalytic Oxygen Reduction**. *Angew. Chem. Int. Ed.* **2021**, *133*, 24346–24354.

(21) Mints, V. A.; Pedersen, J. K.; Bagger, A.; Quinson, J.; Anker, A. S.; Jensen, K. M. Ø.; Rossmeisl, J.; Arenz, M. Exploring the Composition Space of High-Entropy Alloy Nanoparticles for the Electrocatalytic $H_2$/CO Oxidation with Bayesian Optimization. *ACS Catal.* **2022**, *12*, 11263–11271.

(22) Rasmussen, C. E.; Williams, C. K. I. *Gaussian Processes for Machine Learning*; Adaptive Computation and Machine Learning; MIT Press: Cambridge, Mass, 2006.

(23) Jones, D. R.; Schonlau, M.; Welch, W. J. Efficient global optimization of expensive black-box functions. *J. Glob. Optim.* **1998**, *13*, 455–492.

(24) Kushner, H. J. A new method of locating the maximum point of an arbitrary multipeak curve in the presence of noise. **1964**,

(25) Srinivas, N.; Krause, A.; Kakade, S. M.; Seeger, M. Gaussian process optimization in the bandit setting: No regret and experimental design. *arXiv preprint arXiv:0912.3995* **2009**,

(26) Emmerich, M.; Klinkenberg, J.-w. The computation of the expected improvement in

dominated hypervolume of Pareto front approximations. *Rapport technique, Leiden University* **2008**, *34*, 7–3.

(27) Hernández-Lobato, J. M.; Hoffman, M. W.; Ghahramani, Z. Predictive entropy search for efficient global optimization of black-box functions. *Adv. Neural Inf. Process.* **2014**, *27*.

(28) Kraft, D. A software package for sequential quadratic programming. *Forschungsbericht-Deutsche Forschungs- und Versuchsanstalt fur Luft- und Raumfahrt* **1988**,

(29) Virtanen, P. et al. SciPy 1.0: Fundamental Algorithms for Scientific Computing in Python. *Nat. Methods* **2020**, *17*, 261–272.

(30) Hillermeier, C. Generalized homotopy approach to multiobjective optimization. *J. Optim. Theory. Appl.* **2001**, *110*, 557–583.

(31) Clausen, C. M.; Krysiak, O. A.; Banko, L.; Pedersen, J. K.; Schuhmann, W.; Ludwig, A.; Rossmeisl, J. A Flexible Theory for Catalysis: Learning Alkaline Oxygen Reduction on Complex Solid Solutions within the Ag-Pd-Pt-Ru Composition Space**. *Angew. Chem. Int. Ed.* **2023**, *62*, e202307187.

(32) Pedersen, J. K.; Clausen, C. M.; Skjegstad, L. E. J.; Rossmeisl, J. A Mean-Field Model for Oxygen Reduction Electrocatalytic Activity on High-Entropy Alloys**. *ChemCatChem* **2022**, *14*, e202200699.

(33) Rossmeisl, J.; Logadottir, A.; Nørskov, J. K. Electrolysis of water on (oxidized) metal surfaces. *Chem. Phys.* **2005**, *319*, 178–184.

(34) Atkins, P.; De Paula, J.; Friedman, R. *Quanta, matter, and change: a molecular approach to physical chemistry*; Oxford University Press, USA, 2009.

(35) Kulkarni, A.; Siahrostami, S.; Patel, A.; Nørskov, J. K. Understanding Catalytic Activity Trends in the Oxygen Reduction Reaction. *Chem. Rev.* **2018**, *118*, 2302–2312.

(36) Saidi, W. A. Emergence of Local Scaling Relations in Adsorption Energies on High-Entropy Alloys. *npj Comput. Mater.* *8*, 86.

(37) Pedersen, J. K.; Batchelor, T. A.; Yan, D.; Skjegstad, L. E. J.; Rossmeisl, J. Surface Electrocatalysis on High-Entropy Alloys. *Curr. Opin. Electrochem.* *26*, 100651.

(38) Koper, M. T. Thermodynamic theory of multi-electron transfer reactions: Implications for electrocatalysis. *J. Electroanal. Chem.* **2011**, *660*, 254–260.

(39) Stephens, I. E.; Bondarenko, A. S.; Grønbjerg, U.; Rossmeisl, J.; Chorkendorff, I. Understanding the electrocatalysis of oxygen reduction on platinum and its alloys. *Energy Environ. Sci.* **2012**, *5*, 6744–6762.

(40) Greeley, J.; Stephens, I.; Bondarenko, A.; Johansson, T. P.; Hansen, H. A.; Jaramillo, T.; Rossmeisl, J.; Chorkendorff, I.; Nørskov, J. K. Alloys of platinum and early transition metals as oxygen reduction electrocatalysts. *Nat. Chem.* **2009**, *1*, 552–556.

(41) He, Q.; Ding, Z.; Ye, Y.; Yang, Y. Design of high-entropy alloy: a perspective from nonideal mixing. *JOM* **2017**, *69*, 2092–2098.

(42) Zhou, Z.; Zhou, Y.; He, Q.; Ding, Z.; Li, F.; Yang, Y. Machine learning guided appraisal and exploration of phase design for high entropy alloys. *npj Comput. Mater.* **2019**, *5*, 128.

(43) He, Q. F.; Ye, Y. F.; Yang, Y. The Configurational Entropy of Mixing of Metastable Random Solid Solution in Complex Multicomponent Alloys. *J. Appl. Phys.* **2016**, *120*, 154902.

(44) He, Q. F.; Ye, Y. F.; Yang, Y. Formation of Random Solid Solution in Multicomponent Alloys: From Hume-Rothery Rules to Entropic Stabilization. *J. Phase Equilib. Diffus.* **2017**, *38*, 416–425.

(45) Takeuchi, A.; Inoue, A. Classification of bulk metallic glasses by atomic size difference, heat of mixing and period of constituent elements and its application to characterization of the main alloying element. *Mater. Trans.* **2005**, *46*, 2817–2829.

(46) Winter, M. WebElements. https://www.webelements.com/., Accessed 20th Jan. 2023.
